# Cardioprotective and hypotensive mechanistic insights of hydroethanolic extract of *Cucumis melo* L. kernels in isoprenaline-induced cardiotoxicity based on metabolomics and *in silico* electrophysiological models

**DOI:** 10.3389/fphar.2023.1277594

**Published:** 2024-01-29

**Authors:** Muqeet Wahid, Fatima Saqib, Ghulam Abbas, Shahid Shah, Abdulrahman Alshammari, Thamer H. Albekairi, Anam Ali, Muhammad Khurm, Mohammad S. Mubarak

**Affiliations:** ^1^ Department of Pharmacology, Faculty of Pharmacy, Bahauddin Zakariya University, Multan, Pakistan; ^2^ Department of Pharmaceutics, Faculty of Pharmaceutical Sciences, Government College University Faisalabad, Faisalabad, Pakistan; ^3^ Department of Pharmacy Practice, Faculty of Pharmaceutical Sciences, Government College University Faisalabad, Faisalabad, Pakistan; ^4^ Department of Pharmacology and Toxicology, College of Pharmacy, King Saud University, Riyadh, Saudi Arabia; ^5^ School of Pharmacy, Xi’an Jiaotong University, Xi’an, China; ^6^ Department of Chemistry, The University of Jordan, Amman, Jordan

**Keywords:** *Cucumis melo*, cardiac hypertrophy, cardioprotective, metabolomics, myocardial infarction, computational cardiomyocyte simulation, isoprenaline predicted log of inhibition constant (Ki), trifluoroacetic acid (TFA)

## Abstract

**Background:** Cardiovascular diseases (CVD) continue to threaten health worldwide, and account for a significant portion of deaths and illnesses. In both developing and industrialized nations, they challenge their health systems. There are several traditional uses of *Cucurbitaceae* seeds in Pakistan, India, Iran, and China, including treating cardiovascular, neurological, and urogenital diseases.

**Methods:** In the present work, integrated techniques of metabolomics profiling and computational cardiomyocyte stimulation were used to investigate possible mechanisms of *C. melo* in isoprenaline (ISO)-induced myocardial infarction. *In vitro*, vasoconstrictions, paired atria, and *in vivo* invasive blood pressure measurement models were performed to explore the mechanism of action of *C. melo* hydroethanolic seed extract (Cm-EtOH).

**Results:** Results showed that Cm-EtOH demonstrates NO-based endothelium-derived relaxing factor (EDRF) vasorelaxant response, negative chronotropic and inotropic response in the atrium, and hypotensive effects in normotensive rats. Results also revealed that Cm-EtOH decreases cardiomyocyte hypertrophy and reverts the altered gene expressions, biochemical, and metabolites in ISO-induced myocardial infarction (MI) rats. The extract additionally reversed ISO-induced MI-induced oxidative stress, energy consumption, and amino acid metabolism. Moreover, *C. melo* seeds increased EDRF function, energy production, and antioxidant capacity to treat myocardial and vascular disorders. In computational cardiomyocyte simulation, gallic acid reduced action potential duration, upstroke velocity (dV/dt_max_), and effective refractory period.

**Conclusion:** This study highlights the therapeutic potential of *C. melo* seeds to treat cardiovascular diseases and provides mechanistic insight into its antihypertensive and cardioprotective activities.

## 1 Introduction

Cardiovascular diseases (CVDs) continue to threaten health, accounting for a significant portion of deaths and illnesses. Globally, 17.6 million people died from CVD in 2016, compared to 14.5% in 2006 (Benjamin et al., 2019). These complex diseases affect the heart and blood vessels, becoming more prevalent among healthcare professionals (Barbalho et al., 2020). They may include several conditions that are specific but interconnected, including atherosclerosis, stroke, hypertension, peripheral artery disease (PAD), congestive heart failure (HF), cardiac arrhythmias (CA), and coronary artery disease (CAD), which all require specialized treatment (Akter et al., 2022).

Myocardial infarction (MI), known as a heart attack, can permanently damage the heart. The risk of sudden cardiac arrest is increased by 5% because of previous MI, which accounts for 75% of all premature cardiac deaths in modern medicine (Elasoru et al., 2021). During myocardial infarction, cardiac hypertrophy occurs, and irreversible damage to the heart (Benjamin et al., 2019) causes inadequate blood flow and reduces oxygen to the heart tissue (Li et al., 2014). Furthermore, cardiac hypertrophy reduces left ventricular capacity because the left heart ventricle (LHV) becomes abnormally thick. Additionally, the presence of persistent hypertrophy leads to the occurrence of angina, artery atherosclerosis, and heart failure (Lee et al., 2017). When β-adrenergic agonists are upregulated in animal models, they cause heart overload, which leads to myocyte loss, left ventricular (LV) remodeling, and heart failure (Nichtova et al., 2012). ISO (a nonselective agonist) persistently activates the β-adrenergic receptor, which may be used to develop a cardiac hypertrophy and heart failure model in rats (Rau et al., 2017). ISO has often been used to evaluate the pharmacological properties of test drugs or materials for cardioprotection (Nichtova et al., 2012). In complex regulatory systems, several endogenous metabolites and their metabolic pathways can be analyzed using NMR, LC-MS/MS, and GC-MS.

*Cucumis melo Linn*, also called muskmelon, belongs to the Cucurbitaceae family of fruits and vegetables (Duke, 2008). There is widespread cultivation of edible vegetables and fruits of Cucurbitaceae (Mariod et al., 2017; Silva et al., 2020). *Cucumis melo* L. possesses diverse medicinal and nutritional attributes due to its substantial reservoirs of physiologically active compounds. *C. melo* fruits are both nutritious and therapeutic, a juicy, tasty fruit with a sweet taste. The high nutritional content of *C. melo* fruit makes it popular for fresh, canned, or processed consumption. Usually, it is consumed roasted or raw, but it can also be eaten as a salad, dessert, or snack (Erhirhie and Ekene, 2014; Mariod et al., 2017). Melon fruits contain a substantial quantity of antioxidants, ascorbic acid, and carotenoids, in addition to protein and carbs. Seeds are rich sources of vitamin E and omega-3 fatty acids. Melons utilized as vegetables include flavonoids, alkaloids, and bitter constituents, strengthening their potential health-enhancing properties for human consumption (Chang et al., 2015; Gómez-García et al., 2020).

Traditionally, *C. melo* has been used to treat gastrointestinal disorders (Gill et al., 2010) and exhibit antidiabetic (Chen and Kang, 2013), anti-hypothyroidism (Parmar and Kar, 2009), anti-inflammatory, and analgesic activities (Silva et al., 2020) and neurological diseases in Pakistan, India, Iran, and China (Dixit and Kar, 2010; Asif et al., 2014; Patel and Rauf, 2017; Salehi et al., 2019). *C. melo* is traditionally used to reduce blood pressure, anaemia, and dementia (Lim, 2012; Asif et al., 2014). In the Unani medicines system, *C. melo* seeds known as *tukhm-e- Kharbuza* are categorized as “*Muqawwi-e-Qalb*, *Dimagh wa Badan*” which means strengthen the heart, brain, and body (Kirtikar and Basu, 1987; Nadkarni, 1989; Prajapati et al., 2003). *C. melo* seeds are part of Unani cardioprotective formulation “*Muffareh Barid Sada*,” which is recommended to strengthen cardiovascular health (Arzani, 2009; Fahamiya et al., 2016). This unique Unani formulation is a cardiotonic used to treat palpitations or irregular heartbeats. The printed dose of this formulation is 5 g twice a day. Many traditional practitioners in their books recommend using 7–10 g of *C. melo* seeds for medicinal purposes (Rafiquddin, 1985; Hakeem, 2002; Fahamiya et al., 2016).

Furthermore, Yuan et al. (2019) found that *C. melo* exhibited dose-dependent vasodilation in the mesenteric arteries of both mice and rats and suppressed the angiotensin II (Ang II)-induced SBP increase. Due to its effective role in liver steatosis and atherosclerosis (Ibrahim, 2010) it can be an effective medicine for patients with liver disease complicated with hypertension (2019). Similarly, research findings showed that *C. melo* extract exhibits potential anti-atherogenic and cardio-protective benefits in rats with high-fat diet-induced obesity. These effects were believed to be mediated through an antioxidant mechanism and dependence on nitric oxide (Adebayo-Gege et al., 2022).

The anti-angiogenic effect of *C. melo* was studied for purified trypsin inhibitor on three-dimensional cultures of human umbilical vein endothelial cells (Rasouli et al., 2017). Results showed that coumaric and chlorogenic acids significantly prevent cardiovascular disease and cancer (Fanali et al., 2018). Tyrosol and gallic acid can eliminate harmful free radicals, have antibacterial effects against the microorganisms in the gut, and possess anti-inflammatory and anticancer capabilities (Soong and Barlow, 2006). Another study demonstrated the anti-inflammatory, anti-mutation, and growth-inhibiting properties of gallic acid on cancer cells (Jabri-Karoui et al., 2012). Additionally, findings showed that luteolin-7-glycoside, naringenin-7-O-glycoside, and apigenin-7-glycoside possess anti-inflammatory, antioxidant, antitumor, and free radical scavenging characteristics (Bhujbal et al., 2010).

Gallic acid protects cardiovascular diseases (CVDs) by increasing the activity of antioxidant enzymes, inhibiting lipid peroxidation, reducing cardiac marker enzyme levels in the serum, modulating hemodynamic parameters, restoring abnormal electrocardiogram patterns, and preserving histopathological changes. Gallic acid showed a protective effect in different cardioprotective models (Table 1).

**TABLE 1:** Effect of gallic acid on CVDs.

References	Study design	Effects
Priscilla and Prince (2009), Stanely Mainzen Prince et al. (2009); Umadevi et al. (2014); Ryu et al. (2016), Kim et al., (2017)	Isoproterenol-induced cardiac hypertrophy and fibrosis	Decreased ECM proteins (collagens type I and III), CTGF, and TGF-β1
		Decreased CPK, CK-MB, LDH, and cTnT
		Decreased HDAC1 or HDAC2
		Decreased ANP, BNP, and βMHC
		Decreased p-JNKs, p-ERK, and p-Smad3
Ekinci Akdemir et al. (2021)	Doxorubicin-induced myocardial toxicity	Lowered CK-MB, LDH, LDL-c, and VLDL, TG, and increased HDL.
		Increased the Levels of GSH, SOD, and CAT
		Decreased the activity of MDA.
		Elimination of ST segment increase
		Decreased P wave and QRS complex
		Decreased BP
		Decreased histopathological changes
Dianat et al. (2014)	CaCl_2_-induced arrhythmia	Decreased serum levels of CPK, CK-MB, LDH, and cTnT
Jin et al. (2017)	Cardiac hypertrophy and spontaneous hypertension	Decreased cardiac hypertrophy and infarct size
		Modulatory effects on LVP, LVEDP, LVSP, LVDP, and RPP
		Decreased p-JNKs, p- ERK & GATA6 downregulation
Goyal and Patel (2011)	Diabetes-induced myocardial dysfunction	Decreased CPK, CK-MB, LDH, cTnT, and BS.
		Increased SOD, CAT, and GPx
		Increased LVP, LVEDP, LVSP, LVDP, and RPP.
		Increased SOD, CAT, and GPx, decreased the activity of MDA, increase NO.
		Decreased Hypertrophy, infarct size, and Increase vasodilatory response
Jin et al. (2017)	L-NAME-induced hypertension	Decreased incidence VT, VF, and PVB
Dianat et al. (2014)	Cardiac remodeling, and fibrosis	Decreased QT interval prolongation and Increased QRS voltage
	Cirrhosis-induced ECG changes	

In the current study, *C. melo* kernels were investigated *in vivo*, *in vitro*, and molecular docking models for their cardioprotective attributes. Based on the preceding discussion, the present work explores the possible mechanisms of *C. melo* kernels based on serum and heart metabolomic profiling and gene expression using ISO-induced myocardial infarction and computational cardiomyocyte simulation models.

## 2 Materials and methods

### 2.1 Extract preparations

The fruits and seeds of *Cucumis melo* L. were gathered from local farms in Multan, Punjab, Pakistan (Latitude Coordinates: 30.1575°N, 71.5249°E). Dr. Zafar Ullah Zafar, a botanist at Bahauddin Zakariya University, Multan, Pakistan, evaluated, identified, and authenticated the plant. A voucher specimen (Sp. Pl. 1011–1753) was deposited at the university herbarium. Seeds were manually removed from their husks, and an herbal grinder was used to ground the seeds into a coarse powder and then extracted with a hydroethanolic (EtOH; 70:30) solvent in a Soxhlet system at its boiling temperature for 24 h. Then, the solution was filtered, and the extract was concentrated and dried using rotary evaporation at 34°C ± 3°C under reduced pressure to obtain (53% yield) a yellowish-brown greasy product. Dried extracts of *C. melo* seeds (Cm-EtOH) thus obtained were stored in an amber glass jar at −20°C until they were screened for their pharmacological properties. Cm-EtOH was diluted with distilled aqueous or normal saline on experiment day when its pharmacological properties were assessed.

### 2.2 Chemicals

Chemicals and materials used throughout this work were obtained from Sigma Chemicals Co., United States) and were used as received without further purification. Analytical grade solvents ethanol, methanol, acetonitrile, trifluoroacetic acid, and formic acid were procured from Merck & Co., Darmstadt, Germany. Sodium chloride, potassium dihydrogen phosphate, glucose, calcium chloride, sodium biphosphate, potassium chloride, magnesium sulfate, sodium bicarbonate, magnesium chloride, and ethylene tetra-acetic acid were sodium bicarbonate, and ethylene tetra-acetic were procured from Merck & Co, Darmstadt, Germany. Acetylcholine chloride, atropine sulfate, verapamil hydrochloride, and L-NAME were procured from Sigma Chemicals, Co., St. Louis, MO, United States. The analytical grade Apigenin-7-O-Glucoside, Catechin, Chlorogenic acid, Ellagic acid, Epicatechin, Gallic acid, Naringenin, Naringenin-7-O-glucoside, Orientin were procured from Sigma Chemicals, Co., St. Louis, MO, United States.

### 2.3 Phytochemical analysis

The phytochemical characteristics of Cm-EtOH were determined using RP-HPLC and LC ESI-MS/MS following the thresholds and parameters established in our earlier studies (Wahid et al., 2022a; Wahid and Saqib, 2022). The chromatographic samples were prepared in 1 mL of analytical grade methanol (MeOH), centrifuged, and filtered using a syringe filter (0.22 µm).

#### 2.3.1 LC ESI–MS/MS analysis

LC ESI-MS/MS was used to detect the constituents of Cm-EtOH (Wahid et al., 2022c). ESI probes were designed to directly detect positively and negatively charged ions by injecting Cm-EtOH samples into the probes. The binary mobile phase for Cm-EtOH comprised solvent A [0.1% v/v formic acid (FA) in MeOH] and Solvent B [0.1% v/v FA in acetonitrile (ACN) and water (20:80)]. Details of the optimal parameters for chromatography and mass spectrometry can be found elsewhere (Wahid et al., 2022a; Wahid and Saqib, 2022).

#### 2.3.2 RP–HPLC quantification and method validation

The constituents of Cm-EtOH were quantified using RP-HPLC (Wahid et al., 2021; Wahid and Saqib, 2022). The binary mobile phase for Cm-EtOH comprised of solvent A [0.1% v/v trifluoroacetic acid (TFA) in MeOH] and Solvent B [0.1% v/v TFA in ACN and water (20:80)]. Our previous studies have described the details of the optimal parameters for chromatography and the validation of analytical methods (Wahid et al., 2022a; Wahid and Saqib, 2022).

### 2.4 Animal housing and ethical committee provision

The protocols and euthanasia were permitted by the ethical committee of the Department of Pharmacology, Faculty of Pharmacy Bahauddin Zakariya University, Multan (vide No. EC/04PhDL/S2018), and experiments were pursued with the Commission of Laboratory Animal Resources. For *in-vivo* experimental purposes, male Sprague-Dawley rats (♀, weight: 145–160 g 2–3 months old) and for *in-vitro* experiments, Sprague-Dawley rats (♀/♂, weight: 230–280 g 4–5 months old) and for *invasive blood pressure* experiments Sprague-Dawley rats (♀/♂, weight: 200–250 g 3–4 months old) were issued from the Faculty of Pharmacy, Bahauddin Zakariya University, Multan. Animals were kept under control conditions (23°C ± 2°C), in a faculty animal house following a dark and light cycle with standard food and *ad-libitum* tap water. Before the experiment, the feed was removed from animals overnight, but the water had free access. For *in vitro* and *in-vivo* experimentation, rats were deceased with cervical dislocation. The euthanasia of rats was performed in the morning. ♀: male; ♂: female.

### 2.5 Isolated tissue experimentation

The physiological response of the aorta and atrium tissues was recorded using an isometric transducer, following the protocols described in earlier publications (Saqib and Janbaz, 2016; Saqib et al., 2022).

#### 2.5.1 Isolated aorta preparation

In rats, the cervical discs were dislocated, the aorta tissues were excised, and the 3.0 ± 0.5 mm wide aortic rings were obtained. Adhesion of fatty tissues was carefully removed without damaging the endothelial lining of the aorta. The aortic rings were suspended at 37°C ± 0.5°C in a tissue organ bath containing a buffer (pH 7.3) and constantly circulating carbogen under a tension of 2 g. The aortic preparation was stabilized for 55 ± 5 min before Cm-EtOH was administered. Cm-EtOH was evaluated for its vasorelaxant response with potassium chloride (K^+^ 80 mM and K^+^ 25 mM) and phenylephrine (PE; 1 µM)-induced contractions. Calcium-mediated signals are believed to be responsible for smooth muscle contractions, triggering depolarization of muscle fibers by closing potassium ion channels and opening calcium ion channels (Hall and Hall, 2020). Endothelium-derived relaxing factor (EDRF) and muscarinic receptors were examined in denuded and intact aortic endothelium preparations. The aorta rings were rubbed with curved forceps before denuded endothelium aortic tissue preparation. The endothelial functions were determined with phenylephrine and acetylcholine (Ach). The presence of endothelium (intact endothelium) is indicated when Ach (1 µM) relaxes 70%–80% PE (1 µM)-induced contractions. A subsequent increase in contractions was indicative of a denuded endothelium or absence of endothelium in aortic preparations (Kamkaew et al., 2011).

#### 2.5.2 Isolated paired atria

The cervical discs of rats were dislocated, and the heart was excised. The adhesion of fatty tissues and ventricles was carefully removed without damaging the atrium pacemaker. The atrium preparation was suspended at 33°C ± 0.5°C in a tissue organ bath containing a buffer (pH 7.4) and constantly circulating carbogen under a tension of 1 g. The pair atrium preparation was stabilized for 20 ± 5 min before Cm-EtOH was administered.

### 2.6 *In vivo* experimentation

#### 2.6.1 Evaluation of maximum tolerated dose

Cm-EtOH was orally administered to rats for 28 days at 300, 200, 150, 100, and 50 mg/kg per day to determine its maximum tolerated dose; saline was given to control rats. The 28 days study recorded the behavior changes, body weight, mortality, and clinical signs of discomfort (Elasoru et al., 2021).

#### 2.6.2 Determination of hemodynamic and blood pressure parameters in anesthetized rats

We administered intraperitoneally diazepam (5 mg/kg i.p.), and subsequently administrated ketamine (85 mg/kg i.p.) to induce anesthesia in healthy rats (200–250 g) without causing alterations in cardiovascular parameters (Saqib and Janbaz, 2016; Saqib et al., 2022). The rats were supine on the dissection table, and an overhead lamp was used to control the animal temperature. A polyethylene tube (18G) is placed in the trachea during a tracheotomy to ease breathing. We catheterized the right jugular vein to inject Cm-EtOH and other medications intravenously. The heparinized saline (50 IU/mL) filled pressure transducer was catheterized in the left carotid artery to determine the hemodynamic parameters. We applied a saline-soaked cotton swab to the incised area and administered medication after 10–15 min of equilibration. The control hypotension response was obtained with acetylcholine, a cholinergic agonist (1 μg/kg IV), and the control hypertension response was achieved with adrenaline, an adrenergic agonist (1 μg/kg IV).

#### 2.6.3 L-NAME induced acute hypertension

L-NAME, an NO synthase inhibitor (20 mg/kg), was injected intravenously into normotensive rats for an acute antihypertensive study. Cm-EtOH was injected after 10 ± 5 min when L-NAME induced hypertensive rats (Wahid et al., 2022b).

#### 2.6.4 Isoproterenol-induced chronic myocardial infarction

This protocol was designed to induce myocardial infarction (MI) in rats over 21 days following the procedure in an earlier report (Wahid et al., 2022b). Isoproterenol (isoprenaline; ISO) and Cm-EtOH were dissolved in normal saline. In this study, 48 Sprague-Dawley rats (145–160 g) of either sex were randomly divided into different groups.

Group I: as a negative control, normal saline (10 mL/kg) was given orally.

Group II: an intoxicated control continued to receive ISO subcutaneously at a dose of 5 mg/kg/day for 14 days from the 8th day of the experiment to the 21st day with orally administrated normal saline (10 mL/kg).

Group III: positive control, orally received carvedilol (10 mg/kg) for 21 consecutive days.

Group IV: positive control orally received verapamil (10 mg/kg) for 21 consecutive days.

Group V: Low-dose group (LD) orally received Cu.EtOH (75 mg/kg) for consecutive 21 days.

Group VI: High-dose group (HD) orally received Cu.EtOH (150 mg/kg) for consecutive 21 days.

Group VII: extract control group Cu.EtOH at 150 mg/kg orally for 21 consecutive days.

After 1 h of pretreatment of carvedilol, verapamil, and Cm-EtOH, ISO (5 mg/kg/day) was administrated subcutaneously for 14 consecutive days starting from the 8th day until the 21st day, except Group VII, which did not receive the ISO.

#### 2.6.5 Sample collection

A day after the last ISO dose, ketamine (40 mg/kg i.p.) was used to anesthetize the animals. The coagulant and anticoagulant tubes were used to collect blood samples from the retroorbital sinus for analysis and stored at −20°C ± 3°C. Serum and plasma were centrifuged at 4°C at 4,500 rpm for 15 min. Heart tissues were immediately removed from the animals after cervical dislocation and washed with ice-cold normal saline before being frozen for qRT-PCR analysis or fixed in 10% formalin for histological examination.

#### 2.6.6 Determination of biometrical indices

We assessed myocardial tissue hypertrophy using heart diameter, heart weight, heart weight index, tibia length index, tail length index, LHV weight, LHV thickness, and LHV index (Elasoru et al., 2021).

#### 2.6.7 Histopathological examinations

During the autopsy, the hearts were dehydrated in successive alcohol solutions (100%–70%) and fixed in paraffin after being cleaned with xylene. The specimen sections (4 µm) were mounted on glass slides, deparaffinized on a hot plate, and stained with H&E and trichrome stains. The histological changes of myocardial tissues were examined, and all cells’ diameter, size, and total number were quantified; ImageJ (NIH, Version 1.44p) was used to determine these parameters.

#### 2.6.8 Cardiac biochemical markers

In our study, we measured levels of angiotensin II, cGMP, and NO in rats using enzyme immunoassay kits following the manufacturer’s instructions, and commercially available enzyme kits were used to assess biochemical markers of myocardial infarction (Syed et al., 2016).

#### 2.6.9 qRT-PCR

qRT-PCR was conducted in the Bio-Rad CFX96 system to determine relative gene expressions. The frozen heart tissues were extracted with TRIzol reagent as per the manufacturer’s instructions. Listed in Supplementary Table S1 are the primers. We used β-actin as a housekeeping gene to standardize gene expression levels.

#### 2.6.10 Metabolomics study

We conducted the metabolomics analysis according to the procedures outlined by (Sun et al., 2017; Saqib et al., 2022).

##### 2.6.10.1 Sample preparation

Metabolomics study was performed using LC-ESI MS/MS. Serum was precipitated with 400 µL of precooled ACN: MeOH (1:1 v/v) that was vortexed for 60 s at 4°C and then sonicated for 11 ± 2 min at 4°C, followed by overnight incubation at −20°C. The supernatant was then transferred to LC-MS vials after being centrifuged for 15 min at 4°C and 13,000 rpm. The heart tissues were homogenized three times in water (1:10 w/v) and then dried. In this procedure, we mixed dry residue with 400 µL of precooled ACN: MeOH, vortexed for 60 s, sonicated for 11 ± 2 min, centrifuged for 15 min, and collected the supernatant in LC-MS vials.

##### 2.6.10.2 Chromatographic conditions

LC ESI-MS/MS analysis of prepared solutions (8 µL) was performed with binary gradients containing 0.1% FA in MeOH and 0.1% FA in ACN under the chromatographic conditions described in the phytochemical analysis section. The biomarker data were processed as mentioned in our previous publication (Wahid et al., 2022b). MetaboAnalyst was used for functional enrichment analysis.

##### 2.6.10.3 Multivariate data analysis

The multivariate data analysis was performed on serum and cardiac metabolomic using SIMCA 14.0. The metabolic patterns were identified using unsupervised principal component analysis (PCA). The partial least squares discriminant analysis (PLS-DA) was performed to assess and differentiate experimental groups based on the OPLS-DA and PLS-DA methods. A rigorous permutation test was additionally conducted to validate the model. The variable importance parameter (VIP, VIP > 1) and a t-test (*p*-value > 0.05) were performed to screen potential biomarker metabolites.

### 2.7 Molecular docking

Molecular docking was performed using the various modules of Maestro and BIOVIA Discovery Studio 2021 (Wahid et al., 2022a). The LigPrep module was employed to optimize, minimize, and ionize 2D ligands obtained from NCBI PubChem (https://pubchem.ncbi.nlm.nih.gov, accessed 28 August 2021). Protein preparation involves assigning hydrogen bonds, reducing het-states and zero-orders, formation of disulfide bonds, ionizing ions, and optimizing 2D structures. The prime tool was used to fill structural gaps, and the Epik tool was employed to protonate het groups at a pH of 7.0 ± 2.0. PROPKA optimized hydrogen bond arrangements at pH 7.0, and OPLS3e minimized constraint energy. A receptor grid box was generated using the Sitemap module, which determined the coordinates of protein binding pockets and cubic grid boxes. Molecular docking was conducted using prepared ligands, proteins, and receptor grid files. A molecular docking optimization was accomplished, extra precision docking was selected, and the Epik tool was used to apply penalties to the docking score. MM-GBSA was used to calculate the binding energies of Glide ligand-protein complexes. The predicated inhibition constant (Ki) was calculated as previously reported (Wahid et al., 2023).

### 2.8 Computational cardiomyocyte models

We employed the Human LV Endocardium O'Hara-Rudy Model (O’Hara et al., 2011), Human LV heart failure Passini Model (Passini et al., 2016), and chronic RA atrial fibrillation (cAF) Schmidt Model (Schmidt et al., 2015) to simulate the gallic acid response on the electrophysiology of cardiomyocyte according to the method of Sutanto and coworkers (Sutanto et al., 2019). In silico analyses can be performed on healthy and diseased conditions involving either pharmacological inhibition of ion channels or no inhibition.

### 2.9 Software and statistical analysis

GraphPad Prism 8 and RStudio (v 2021.02.3) were used for statistical analysis. In this study, we calculated the median effective concentration (EC_50_) using a sigmoidal dose-response curve fitted with a nonlinear regression equation based on the mean and standard deviation (SD). The dose-response curves were constructed using logarithmic sigmoidal dose-response graphs. For the *in vivo* study, one-way ANOVA, Dunnett test, and Student’s t-test were used, with *p* < 0.05 considered significant. Biorender (https://biorender.com/) and Adobe Illustrator were used to draw the illustration figures of the mechanism. LabChart Pro 7 was used for physiological recordings, Maestro 12.8 (Schrodinger Inc., United States) to perform and visualization of molecular docking, and BIOVIA Discovery Studio for compound-amino acid interaction data.

## 3 Results

### 3.1 Identification of bioactive compounds

Results of our study of the bioactive compounds in Cm-EtOH using LC ESI–MS/MS showed the presence of ten phytochemicals. Shown in Supplementary Table S2 are the fragmentations of phytochemical constituents.

### 3.2 HPLC method validation and parameter optimization

The binary mobile phase system at a flow rate of 0.8 mL per minute was employed to separate the phytoconstituents of Cm-EtOH. HPLC parameters were adjusted to separate Cm-EtOH with complete elucidation and distinct peaks. The separation of peaks of Cm-EtOH was improved by using 320, 280, and 250 λ nm wavelengths, and peaks were compared with external standards in this study. To validate the HPLC method, linearity, recovery, and instrument precision on intra-day and inter-day were evaluated with calibration curves for diluted external standards (Table 2; Supplementary Tables S3, S4).

**TABLE 2:** Bioactive compounds quantified and validated in *C. melo* L seeds by HPLC DAD-UV/Vis.

Analytes	λ (nm)	Rt (mins)	Linear Regression data			LOD (µg/mL)	LOQ (µg/mL)	Concentration (µg/g)	Precision (RSD %)		Recovery		Analytes + extract (µg/g)	
			Range (µg/mL)	Equation	r^2^				Inter day	Intra day	Mean	RSD%	50 µg	100 µg
β-Sitosterol	250	17.6	7.81–500	y = 165.27x + 17.29	0.9997	0.35	1.07	214.68	0.52	0.94	99.01 ± 0.74	0.74	263.21	314.03
Chlorogenic acid	280	5.1	7.81–500	y = 153.24x + 29.37	0.9999	0.53	1.61	471.12	0.79	1.82	98.84 ± 1.89	1.91	520.43	570.37
Protocatechuic acid		9.4	7.81–500	y = 112.47x + 19.68	0.9996	0.51	1.54	343.15	0.69	1.62	98.80 ± 0.89	0.90	392.81	441.91
Orientin		16.4	7.81–500	y = 172.45x + 25.45	0.9999	0.44	1.32	561.56	0.61	1.34	99.32 ± 1.34	1.35	610.25	660.73
Gallic acid	320	3.3	7.81–500	y = 193.84x + 21.12	0.9998	0.64	1.94	481.63	1.56	1.77	98.50 ± 0.99	1.01	529.87	580.93
Vitexin		18.4	7.81–500	y = 213.83x + 25.65	0.9999	0.56	1.71	497.78	0.84	1.63	98.66 ± 1.78	1.80	546.31	597.01

### 3.3 Quantification of phytochemicals in Cm-EtOH

The linear regression model was used to quantify phytoconstituents based on calibration curves from external standards. Shown in Table 2 and Supplementary Figure S1 are the quantification of phytoconstituents of Cm-EtOH.

### 3.4 Vasorelaxant effects on rat aortic preparations

**FIGURE 1:**
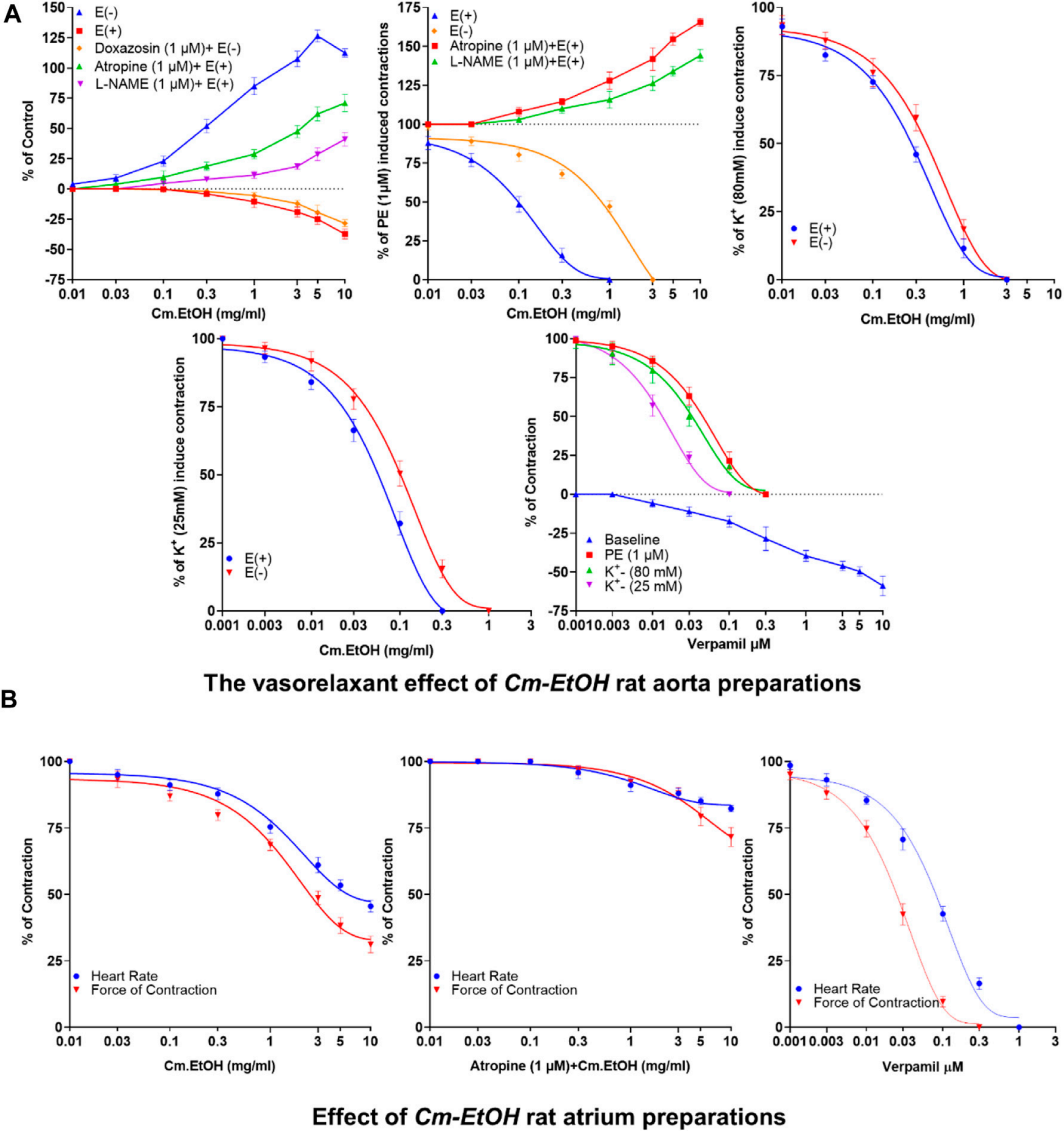
Vasorelaxant and the cardiac response of verapamil and Cm-EtOH on isolated endothelial intact and denuded aortic and paired atrium preparations. **(A)** Cm-EtOH and verapamil exert vasorelaxant effects on baseline and spastic contractions of PE (1 µM), K^+^ (80 mM), and K^+^ (25 mM) in intact and non-intact (denuded) aortic preparations. **(B)** Cm-EtOH and verapamil exert negative inotropic and chronotropic paired atriums. A sigmoidal dose–response curve was used to analyze values, data presented as the mean ± SD, *n* = 5.

Depicted in Figure 1A are our results of the vasorelaxant effects of Cm-EtOH extract on endothelial intact and denuded aortic preparations. Our findings revealed that Cm-EtOH exerts vasorelaxant properties in preparing an aorta with an intact endothelium without causing any vasoconstriction. However, Cm-EtOH produced vasoconstriction in endothelium-intact aortic tissue upon pretreatment with L-NAME (1 µM) and atropine (1 µM). Furthermore, Cm-EtOH caused vasoconstriction in denuded aortic preparations and partial vasorelaxation at 5 and 10 mg/mL doses. In contrast, doxazosin (1 µM) inhibited these constrictions and relaxed denuded aortic preparations, whereas Cm-EtOH reversed PE (1 µM), K^+^ (80 mM), and K^+^ (25 mM) elicited vasoconstriction in endothelial intact and denuded aortic preparations. In addition, our findings indicated that PE (1 µM), K^+^ (80 mM), and K^+^ (25 mM) have EC_50_ values of 2.174 (1.011–6.719), 0.5835 (0.2619–0.468), and 0.1199 mg/mL (0.09644–0.1492) at 95% CI in denuded aortic preparations, and have EC_50_ values of 0.1201 (0.8093–0.1780), 0.3495 (0.2619–0.4685), and 0.08741 mg/mL (0.06657–0.1163) in intact endothelium. Despite this, Cm-EtOH failed to relax PE (1 µM)-induced contractions in endothelium-intact aortic preparations pretreated with L-NAME (1 µM) and atropine (1 µM). Verapamil was compared to these results to determine whether Cm-EtOH might have calcium-antagonistic properties. In aortic preparations, verapamil attenuated PE (1 µM), K^+^ (80 mM), and K^+^ (25 mM)-induced constrictions, with EC_50_ values of 0.06180 (0.04533–0.08532), 0.03777 (0.02496–0.05796), and 0.01415 µM (0.01022–0.01964), respectively.

### 3.5 Effect of Cm-EtOH on isolated rat paired atria preparations

The inotropic and chronotropic effects of Cm-EtOH were investigated in rat-paired atria (Figure 1B). Results showed that Cm-EtOH reduces heart rate and force of contraction with EC_50_ values of 1.817 (1.221–2.736) and 1.561 mg/mL (0.9762–2.51) at 95% CI. Thus, Cm-EtOH exerts a negative inotropic and chronotropic effect on the paired atrium. Pretreatment with atropine (1 µM) reduced the negative chronotropic and inotropic effects of Cm-EtOH. In contrast, verapamil caused an inhibitory response to heart rate and force of contraction with EC_50_ values of 0.09041 (0.07194–0.1137) and 0.2947 µM (0.02177–0.04006), respectively.

### 3.6 Maximum tolerated doses of *C. melo* seed extracts

Cm-EtOH was tested on rats to determine its maximum tolerable dose. Our findings showed no rat deaths, behavioral abnormalities, weight changes, or clinical signs of distress or discomfort during the 28-day test period.

### 3.7 Effect Cm-EtOH on blood pressure and hemodynamic parameters

**FIGURE 2:**
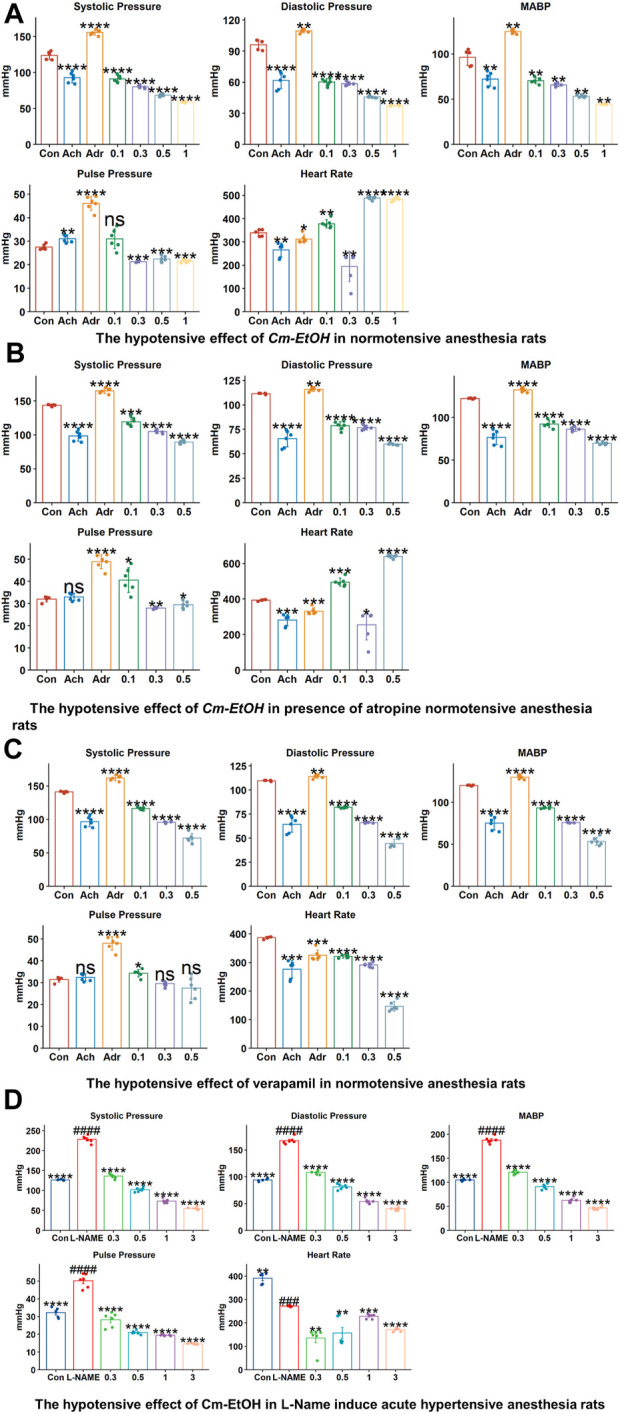
The hypotensive effects of verapamil and Cm-EtOH on normotensive and L-NAME induced acute hypertensive rats. **(A)** The hypotensive effects of Cm-EtOH on normotensive rats. **(B)** The hypotensive effect of Cm-EtOH on normotensive rats in the presence of atropine (1 mg/kg). **(C)** The hypertensive effect of verapamil on normotensive rats. **(D)** The hypotensive effect of Cm-EtOH on L-NAME (20 mg/kg)-induced acute hypertensive rats. Student t-tests were applied and compared to the control and L-NAME; *p* < 0.05 was considered a significant value. Data represented as the mean ± SD. *n* = 5, (****p < 0.0001, ***p < 0.001, **p < 0.01, and *p < 0.05, versus the control; ^####^ *p* < 0.0001, ^###^ *p* < 0.001, ^##^ *p* < 0.01, and ^#^ *p* < 0.05, versus L-NAME). Adr, adrenaline; Ach, acetylcholine; Con, Control.

To study the effect of the extract on blood pressure and hemodynamic parameters, Cm-EtOH was administered to normotensive rats to measure their systolic and diastolic blood pressure. Results revealed that Cm-EtOH dose-dependently reduces the pulse pressure (PP), systolic (SBP), diastolic (DBP), mean arterial blood pressure (MABP), and heart rate as shown in Figure 2. Moreover, when Cm-EtOH was administered at 0.3, 0.5, and 1 mg/kg doses, it reduced the MABP to 65.70 ± 1.79, 53.28 ± 1.20, and 44.4 ± 0.43 mmHg, respectively as depicted in Figure 2A. However, atropine (1 mg/kg) attenuated this hypotensive response of Cm-EtOH with MABP 69.81 ± 1.57 mmHg at 1 mg/kg dose (Figure 2B). On the other hand, verapamil decreased the MABP by 93.34 ± 0.91, 75.65 ± 4.66, and 55.62 ± 4.63 mmHg at 1, 3, and 10 μg/kg doses (Figure 2C). The MABP of control, acetylcholine, and adrenaline were 118.03 ± 0.66, 74.08 ± 6.15, and 134.91 ± 1.24 mmHg, respectively.

### 3.8 L-NAME induced acute hypertension

L-NAME was administered to anesthetized normotensive rats to cause acute hypertension, thus increasing MABP (193.27 ± 5.25 mmHg). Cm-EtOH significantly reduced the MABP of hypertensive rats to 126.35 ± 5.73, 73.91 ± 2.01, and 61.11 ± 2.44 mmHg at 0.3, 0.5, and 1 mg/kg doses (Figure 2D).

### 3.9 Cardioprotective effects of Cm-EtOH on isoprenaline-induced chronic myocardial infarction

In this study, biometrical, histopathological, and biochemical parameters were used to assess the effects of Cm-EtOH on isoprenaline (ISO) induced chronic myocardial damage. Our findings indicated that Cm-EtOH protects rats from ISO-induced chronic myocardial damage.

#### 3.9.1 Survival rate and physical activity

Myocardial infarction (MI) was strongly associated with the survival rate and levels of physical activity. Our findings showed no fatalities in the verapamil, carvedilol, Cm-EtOH (75 mg/kg), and Cm-EtOH (150 mg/kg) groups; however, there were fatalities in the ISO group. The subcutaneous administration of ISO reduced physical activity on the fourth dose, and the tenth dose caused a complete loss of physical activity with shortness of breath. It was observed that animals in the verapamil, carvedilol, and Cm-EtOH (150 mg/kg) group show minimal shortness of breath and were active. Several other metrics, including exhaustion, breathlessness, physical activity, and weight loss increased in the ISO group. In contrast, a change in weight or other measurements was not observed in verapamil, carvedilol, and Cm-EtOH (150 mg/kg) groups.

#### 3.9.2 Effect on histopathological parameters

**FIGURE 3:**
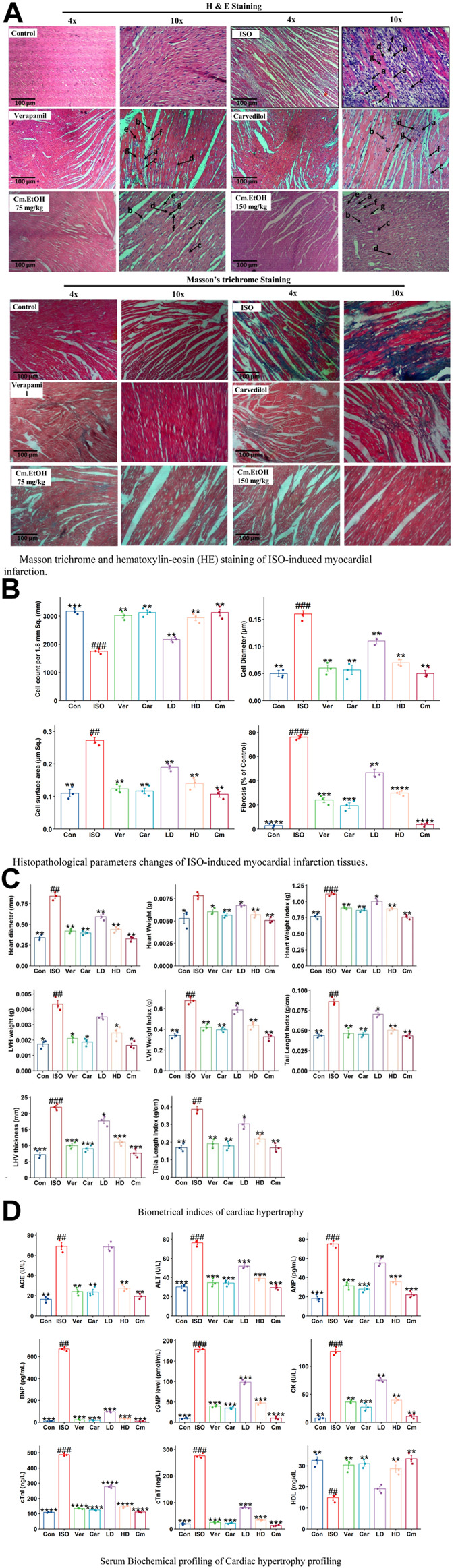
Histomorphology alterations in ISO-induced myocardial infarction tissues, cardiac hypertrophic and serum biochemical changes in of ISO, carvedilol (10 mg/kg), verapamil (10 mg/kg), and Cm-EtOH (75 and 150 mg/kg). **(A)** Masson trichrome and hematoxylin-eosin (HE) staining of ISO-induced MI myocardial tissues. **(B)** Histopathological parameters changes of ISO-induced MI myocardial tissues. a) Necrotic myocardial fibers; b) Macrophages; c) neutrophil infiltrations; d) Interstitial edema; e) mucoid degeneration; f) fibroblast; g) loss of striations with nuclear changes. **(C)** Effect on cardiac hypertrophic parameters in ISO-induced myocardial infarction tissues. **(D)** Effect on serum biochemical parameters of ISO-induced MI rats. Student’s t-tests were applied and compared to the ISO, whereas ISO was compared to the control; *p* < 0.05 was considered a significant value. Data represented as the mean ± SD. n = 6, (****p < 0.0001, ***p < 0.001, **p < 0.01, and *p < 0.05, vs. ISO; ####*p* < 0.0001, ###*p* < 0.001, ##*p* < 0.01, and #*p* < 0.05, versus the control). Con: Control, Car: Carvedilol (10 mg/kg); ISO: isoproterenol (5 mg/kg/day), Ver: Verapamil (10 mg/kg); LD: Low Dose (75 mg/kg); HD: High-Dose (150 mg/kg); Cm: Cm-EtOH (150 mg/kg).

The histologic examinations of tissues were conducted to identify the extent of necrosis in cardiac tissues by examining inflammatory cells and fibroblasts (Figure 3A). The diameter, thickness, and number of cells were measured. Findings from this study showed a significant increase in collagen hyperplasia, inflammation, and interstitial edema in the myocardial tissue in the ISO group. This tissue was infiltrated with neutrophils, had poor myofibers, pyknosis, and karyorrhexis, as well as structurally disorganized fibrous tissue (Figure 3A). Our results indicated that Cm-EtOH substantially reduced inflammation and fibrosis without significant myocardial tissue damage, except for low-dose Cm-EtOH (75 mg/kg) which failed to protect animals. In addition, results revealed that Cm-EtOH, verapamil, and carvedilol at high doses reduce necrobiosis, which increases cell count without reducing cell diameter or surface area (Figure 3B), suggesting that Cm-EtOH protects rats from ISO-induced myocardial infarction.

#### 3.9.3 Effects on biometrical indices

Figure 3C illustrates the effects of Cm-EtOH effects on the biometric indicators of ISO-induced chronic cardiac hypertrophy. The subcutaneous administration of ISO was found to raise the biometrical indicators of cardiac hypertrophy in the disease group (ISO) including heart diameter, heart weight, heart weight index, LHV weight and thickness, tibia length index, tail length index, and LHV index. However, carvedilol, verapamil, and Cm-EtOH (150 mg/kg) reduced these biometrical indicators and protected the animals from cardiac hypertrophy. There was significant protection against cardiac hypertrophy in the treated groups compared to animals in the ISO group. Furthermore, there was no significant difference between the treated and control groups. Cm-EtOH extract provides minimal protection for animals at 75 mg/kg dose. Physical assessment revealed that the ISO group has significantly thicker LHV than the control group which indicates a higher incidence of hypertrophy, whereas the verapamil, carvedilol, and Cm-EtOH (150 mg/kg) pretreated groups did not exhibit any noticeable thickness.

#### 3.9.4 Effects on serum cardiac biochemical markers

Myocardial infarctions are clinically associated with several serum biochemical indicators, including ANP, CK-MB, CK, cTnI, cTnT, LDH, and lipid profiles. Furthermore, tests such as NO, ACE, AST, ALT, and renin can be used to assess cardiac injury, along with serum levels of cGMP, IL-6, and BNP. The biomarkers of the control group were found to differ significantly from the ISO group (Figure 3D; Supplementary Figure S1). Our findings showed that serum biomarkers for verapamil, carvedilol, and Cm-EtOH groups were within acceptable limits. However, in the ISO and Cm-EtOH (75 mg/kg) groups, these markers were elevated. In this study, it was demonstrated that Cm-EtOH extract prevented the cardiotoxicity induced by ISO in rats.

#### 3.9.5 Gene expression analysis

**FIGURE 4:**
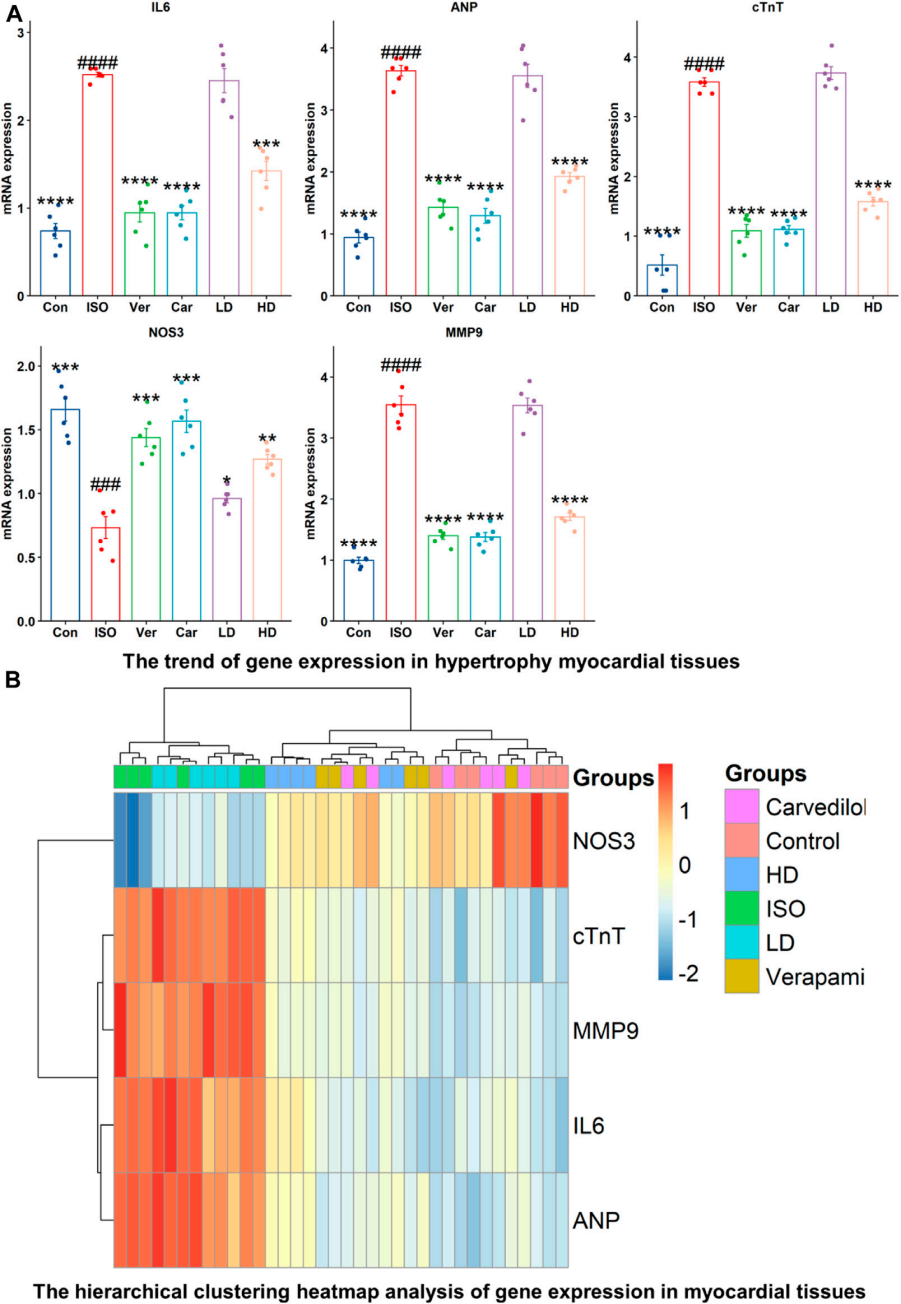
RNA expression levels of NOS3, cTnT, MMP9, IL-6, and ANP in ISO-induced myocardial infarction tissues of ISO, Carvedilol, Verapamil, Cm-EtOH. **(A)** Effect of Cm-EtOH, carvedilol, and verapamil on RNA levels in ISO-induced myocardial tissues. **(B)** Expression of NOS3, cTnT, MMP9, IL-6, and ANP in hierarchical clustering heatmap; Red: increase in RNA expressions; Blue: decrease in RNA expressions. Student t-tests were applied and compared to ISO, whereas ISO compared to the control; *p* < 0.05 was considered a significant value. Data are given as the mean ± SD. *n* = 6, (****p < 0.0001, ***p < 0.001, **p < 0.01, and *p < 0.05, vs. ISO; ^####^ *p* < 0.0001, ^###^ *p* < 0.001, ^##^ *p* < 0.01, and ^#^ *p* < 0.05, versus the control). Con: Control, Car: Carvedilol (10 mg/kg); ISO: isoproterenol (5 mg/kg/day), Ver: Verapamil (10 mg/kg); LD: Low Dose (75 mg/kg); HD: High-Dose (150 mg/kg).

Inflammation and cardiovascular disease are associated with expressions of ANP, cTnT, NOS3, MMP-9, and IL-6 genes (Figure 4). The mRNA expressions of ANP, cTnT, MMP-9, and IL-6, were decreased significantly in verapamil, carvedilol, and Cm-EtOH (150 mg/kg) pretreated groups; however, mRNA expressions of these genes were increased in the ISO group. Results showed that Cm-EtOH (75 mg/kg) provides a low level of protection for animals (Figure 4A). However, carvedilol, verapamil, and Cm-EtOH (150 mg/kg) markedly increased NOS3 mRNA expression. In contrast, its expression was significantly reduced in hypertrophied hearts. The normalized levels of gene expression were determined by conducting a hierarchical clustering heatmap analysis of ISO, control, carvedilol, verapamil, and Cm-EtOH as shown in Figure 4B. Except for Cm-EtOH (75 mg/kg), results indicate that treated group expressions were significantly below the ISO group expressions while similar to the control group. These findings demonstrated that Cm-EtOH (150 mg/kg) alters and restores the trend of gene expressions like the carvedilol and verapamil groups.

#### 3.9.6 Metabolomics profiling analysis

##### 3.9.6.1 Multivariate data analysis

**FIGURE 5:**
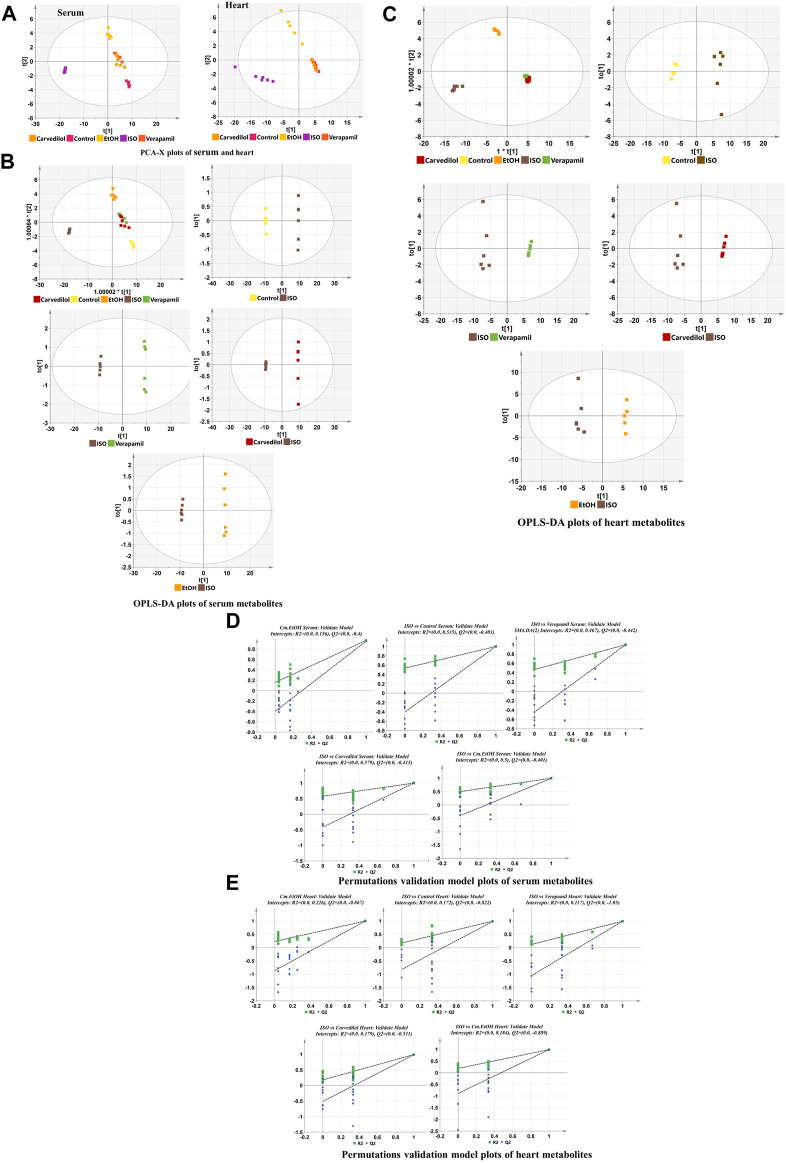
The Discrimination model plots of heart tissue and serum metabolites in ISO-induced myocardial infarction tissues of ISO, carvedilol, verapamil, and Cm-EtOH. **(A)** PCA-X. OPLS-DA plots of **(B)** heart tissue and **(C)** serum metabolites. The permutation validation plots of **(D)** serum and **(E)** heart tissue metabolites in ISO-induced myocardial infarction rats of ISO, Carvedilol (10 mg/kg), Verapamil (10 mg/kg), and Cm-EtOH (75 and 150 mg/kg).

The metabolome data were evaluated using principal component analysis. The unsupervised PCA plots of heart metabolomes (R^2^X = 0.866, Q^2^ = 0.829) and serum metabolomes (R^2^X = 0.906, Q^2^ = 0.893) separated the ISO group from the other groups, including carvedilol, verapamil, and Cm-EtOH (150 mg/kg) (Figure 5A). Our findings showed a considerable metabolic change in the ISO group that successfully predicts the validation of the ISO-induced MI model. We used the supervised OPLS-DA plot to distinguish metabolites of ISO, carvedilol, verapamil, and Cm-EtOH (150 mg/kg) in the serum (Figure 5B) and heart (Figure 5C). OPLS-DA plots revealed significant differences between ISO and carvedilol, verapamil, and Cm-EtOH (150 mg/kg). However, carvedilol, verapamil, and Cm-EtOH did not differ significantly. Furthermore, there was an intersection between carvedilol, verapamil, and Cm-EtOH groups. OPLS-DA models were found to be fit and predictable without being overfitted according to results with significant increases in R^2^X, Q^2^, and CV-ANOVA values. These OPLS-DA models consistently showed a high level of accuracy and stability for serum (R^2^X = 0.976, Q^2^ = 0.975) and myocardium tissues (R^2^X = 0.993, Q^2^ = 0.99). Results from this study indicated that ISO, carvedilol, verapamil, and Cm-EtOH (150 mg/kg) show significant differences in metabolite concentrations based on the OPLS-DA analysis. OPLS-DA models were also used to compare serum and cardiac metabolites between ISO and treated groups as shown in Supplementary Table S5.

In addition, the permutation test (Figures 5D, E) with cross-validation was used to confirm the statistical validity of serum and cardiac tissue metabolites in the Cm-EtOH (150 mg/kg), verapamil, carvedilol, and ISO groups. With the negative intercept of Q^2^, the model is highly predictive and highly correlated due to its predictability, overlap, and correlation between permuted and original data. Permutation tests indicate that the models are stable and explain variations in cardiac tissue (R^2^ = 0.139, Q^2^ = −0.198) and serum (R^2^ = 0.17, Q^2^ = −0.395).

##### 3.9.6.2 Analysis of the biomarkers in different samples

**FIGURE 6:**
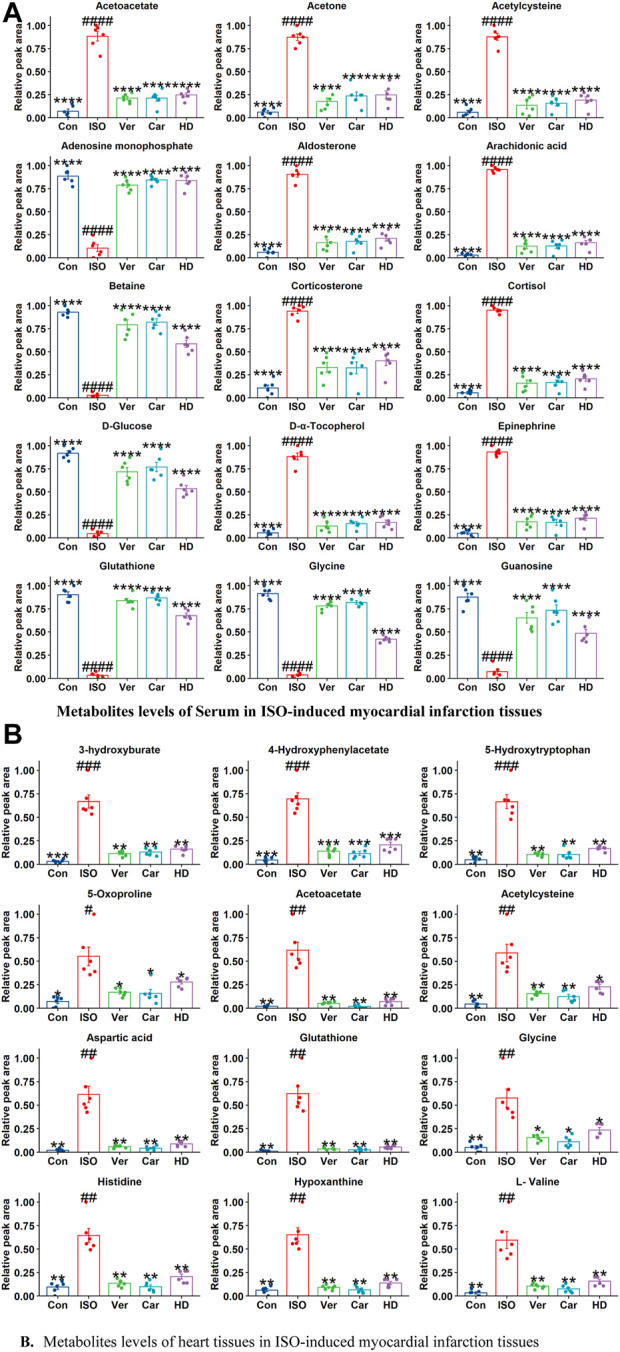
The metabolites levels of **(A)** serum and **(B)** heart tissues in ISO-induced myocardial infarction rats of ISO, Carvedilol, Verapamil, Cm-EtOH. Student’s t-tests were applied and compared to the ISO; whereas ISO compared to the control; *p* < 0.05 was considered a significant value. Data are presented as the mean ± SD. *n* = 6, (****p < 0.0001, ***p < 0.001, **p < 0.01, and *p < 0.05, vs. ISO; ^####^ *p* < 0.0001, ^###^ *p* < 0.001, ^##^ *p* < 0.01, and ^#^ *p* < 0.05, versus the control). Con: Control, Car: Carvedilol (10 mg/kg); ISO: isoproterenol (5 mg/kg/day), Ver: Verapamil (10 mg/kg); LD: Low Dose (75 mg/kg); HD: High-Dose (150 mg/kg); Cm: Cm-EtOH (150 mg/kg).

Metabolome data were used to determine metabolic pathways and metabolites linked to MI in a metabolomics study. A total of 63 metabolites were found in cardiac tissue, whereas 73 were found in the serum. These metabolites include glycoproteins, betaine, mineralocorticoids, amino acids, ketones, organic acids, eicosanoids, choline, glycerol, and glucose. In heart tissue, a total of 63 metabolites were found. Figure 6; Supplementary Figures S3, S4 illustrate the changes in metabolites in serum and heart tissues. Results show a significant difference between rats in the ISO group and those treated with carvedilol, verapamil, and Cm-EtOH (150 mg/kg). In the ISO group, altered metabolites from the control in serum and heart tissues indicate myocardial infarction. However, treatment of rats with carvedilol, verapamil, and Cm-EtOH (150 mg/kg) significantly normalized the concentrations of the metabolite and prevented ISO-induced MI in rats.

##### 3.9.6.3 Metabolic pathway analysis

**FIGURE 7:**
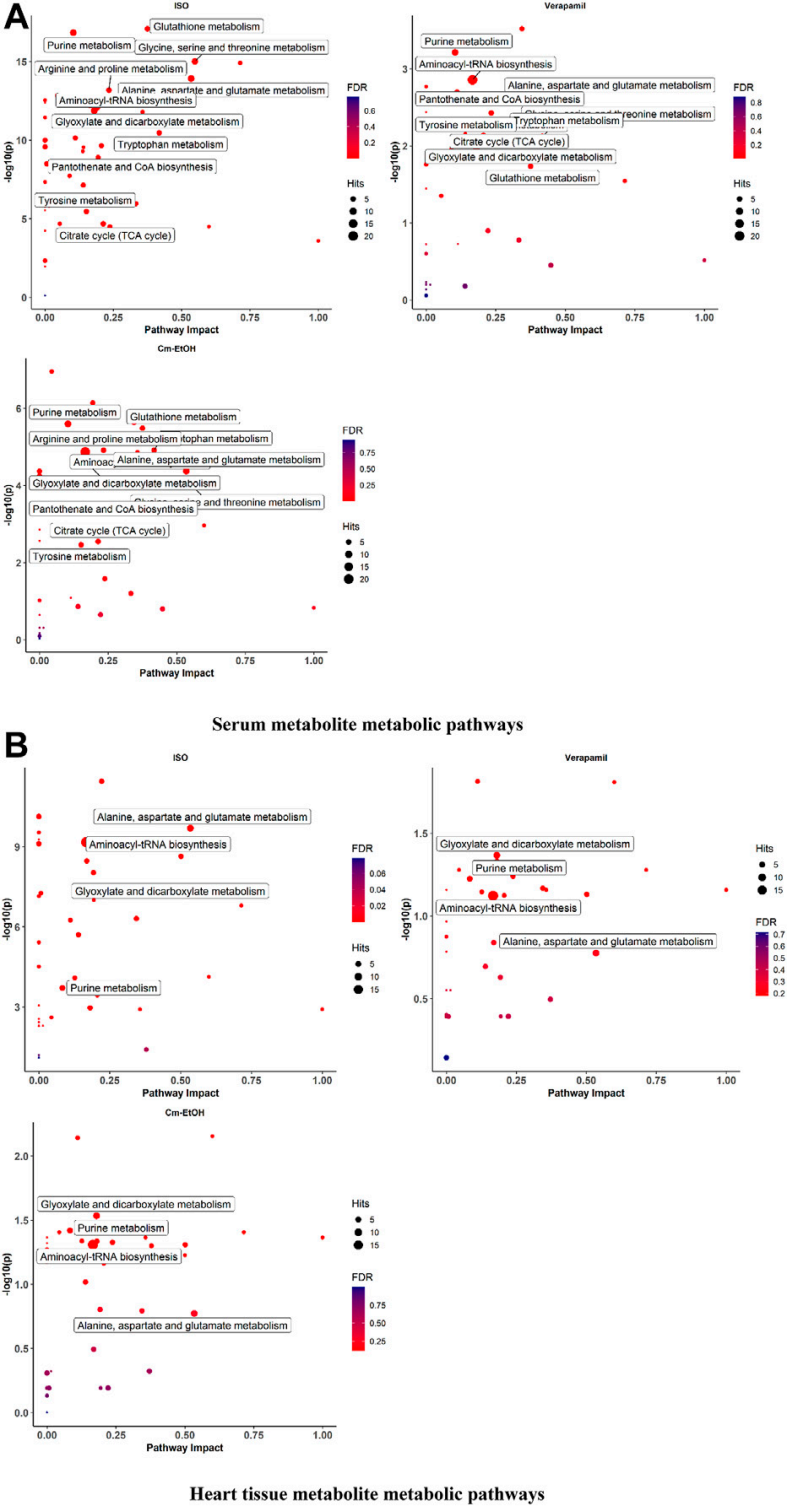
The metabolite metabolic pathways change in **(A)** serum and **(B)** heart in ISO-induced myocardial infarction rats of ISO, Carvedilol, Verapamil, Cm-EtOH (75 mg/kg), and Cm-EtOH (150 mg/kg).Data were normalized using a control group.

We employed MetabolAnalyst to analyze metabolites for the identification of the metabolic pathways enriched by myocardial infarction as shown in Figure 7. Our findings indicate noticeable changes in oxidative stress, energy metabolism, and amino acid metabolism in the serum and cardiac metabolites. The ISO group showed alterations in several metabolic pathways and higher levels of certain metabolites. In this respect, it is worth mentioning that verapamil and Cm-EtOH (150 mg/kg) moderated metabolic pathways to prevent myocardial damage. In contrast, the ISO group demonstrated significant increases in BCAA, glutamine, glutamate, ketone bodies, and amino acid metabolism, which were reversed by Cm-EtOH (150 mg/kg) and verapamil. Thus, Cm-EtOH (150 mg/kg) exerted beneficial effects on the heart in studies examining metabolic pathways.

##### 3.9.6.4 Correlation and heatmap analysis of metabolites

**FIGURE 8:**
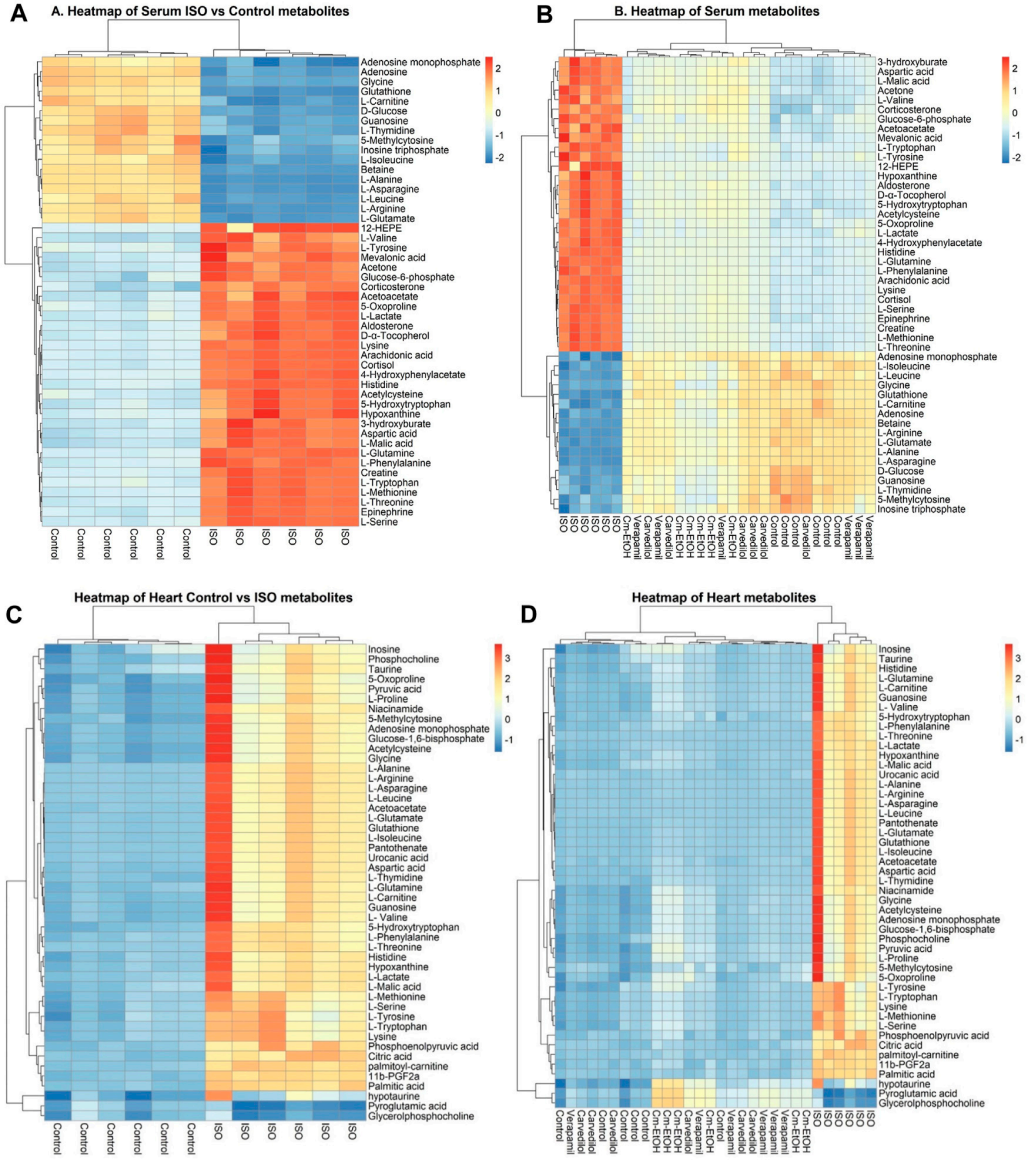
Heterarchical clustering heatmap for metabolite alteration after ISO induces MI. **(A)** Comparison of serum metabolites between control and ISO groups, **(B)** Comparison of serum metabolites between control, ISO, verapamil, carvedilol, and Cm-EtOH (150 mg/kg) groups. **(C)** Comparison of heart metabolites between control and ISO groups, **(D)** Comparison of heart metabolites between control, ISO, verapamil, carvedilol, and Cm-EtOH (150 mg/kg) groups.

The correlation and heatmap techniques were used to determine the significance of Cm-EtOH. Pearson correlation coefficients were used to determine the relationship between the altered metabolites. Red indicates that there is a positive correlation, while green signifies that there is a negative correlation (Supplementary Figure S5). We performed a hierarchical clustering heatmap analysis on the control, ISO, carvedilol, verapamil, and Cm-EtOH (150 mg/kg) to determine normalized intensities of endogenous metabolites. The difference in the concentrations of metabolites was found between the treated and ISO groups. However, the concentrations of metabolites in treated groups were like the control group. A rise in serum metabolites is depicted in red, whereas a fall is seen in blue (Figure 8).

### 3.10 Molecular docking

**FIGURE 9:**
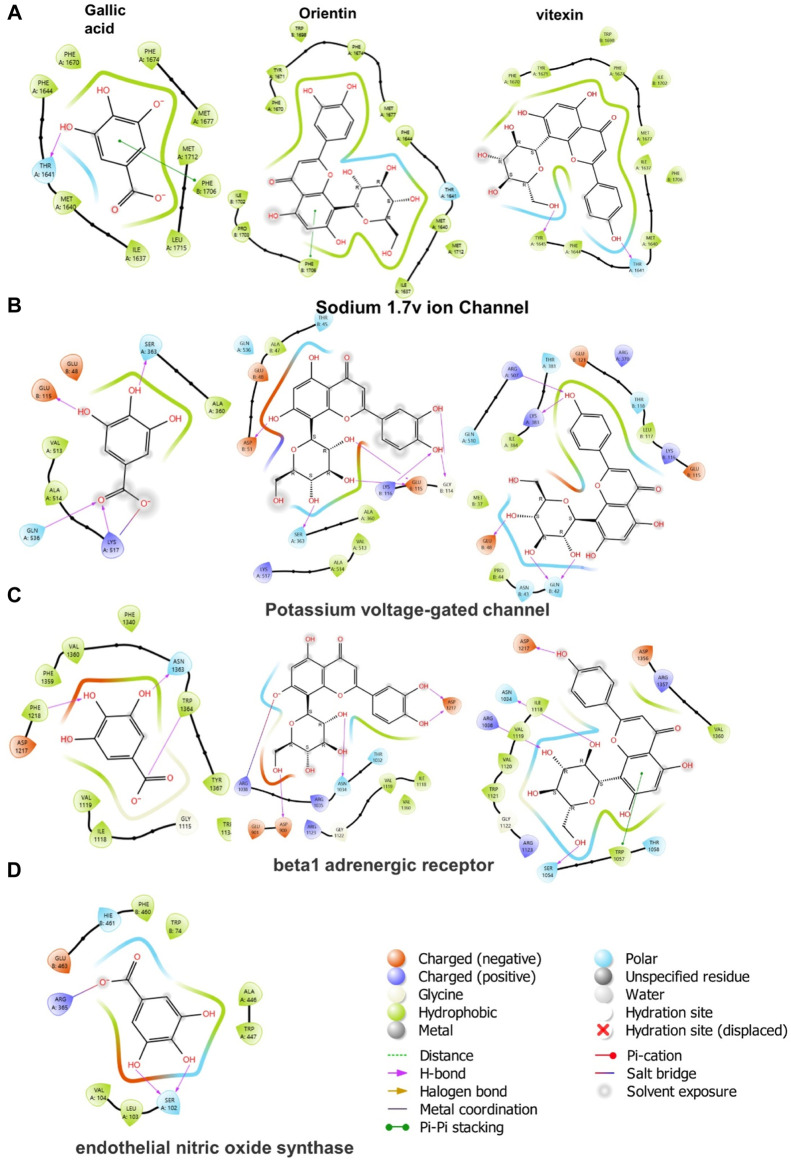
2D ligand-protein interactions between Cm-EtOH compounds and proteins; **(A)** Human sodium channel voltage-gated ion channel (Nav1.7 PDB: 5EK0) **(B)** Potassium voltage-gated ion channel (KCNQ1; PDB: 5VMS) **(C)** Human β-1 adrenergic receptor (PDB: 7BTS) **(D)** Human endothelial nitric oxide synthase (eNOS; PDB: 1M9J).

In molecular docking, it is possible to predict the location of a ligand in a protein. The precision of docking models is enhanced by an appropriate force field and physical energy components (Sirous et al., 2019; Wahid et al., 2022c). In the docking experiment, the human endothelial nitric oxide synthase (eNOS; PDB: IM9J), PDB: human potassium voltage-gated ion channel (KCNQ1; PDB: 5VMS), human sodium channel voltage-gated ion channel (Nav1.7 PDB: 5EK0), and human β-1 adrenergic receptor (PDB: 7BTS) were docked with vitexin, orientin, and gallic acid to show the positive effect target genes; results are shown in Table 3 and Figure 9.

**TABLE 3:** Docked ligand–protein complex binding energies (kcal/mol) calculated with Prime MM–GBSA.

Compounds	Docking score (kcal/mol)	Glide energy (kcal/mol)	∆G bind (kcal/mol)	Log pKi (µM)	∆G Coulomb (kcal/mol)	∆G Covalent (kcal/mol)	∆G Hbond (kcal/mol)	∆G Lipo (kcal/mol)	∆G Packing (kcal/mol)	∆G Solv GB (kcal/mol)	∆G vdW (kcal/mol)	Residue–Ligand interactions with distance (Å)	
												Hydrogen bonds	Electrostatic/Hydrophobic bonds
Human endothelial nitric oxide synthase (eNOS; PDB: 1M9J)													
Gallic acid	−7.152	−25.536	−17.49	−4.37	−62.46	4.92	−2.26	−10.44	−3.26	80.67	−24.66	Conventional Hydrogen Bond: Trp447 (2.81), Ser102 (1.89), Ser102 (1.69), Carbon Hydrogen Bond: His461 (2.69)	Salt Bridge; Attractive Charge: Arg365 (2.34), Pi-Anion: Trp74 (4.93), Pi-Pi Stacked: Trp447 (5.42), Trp447 (5.31), Pi-Alkyl: Val104 (5.06)
Human sodium channel voltage-gated ion channel (Nav1.7 PDB: 5EK0)													
Vitexin	−9.762	−41.855	−57.1	−21.57	−31.48	0.15	−1.09	−13.06	−4.85	25.9	−32.66	Conventional Hydrogen Bond: Tyr1645 (1.85). Thr1641 (1.90). Carbon Hydrogen Bond: Tyr1671 (3.08). Tyr1671 (2.49)	Pi-Pi Stacked: Trp1698 (4.60). Pi-Alkyl: Ile1702 (5.18)
Orientin	−9.208	−32.962	−40.8	−14.49	−10.7	5.38	−0.51	−20.32	−4.25	23.52	−33.92	Conventional Hydrogen Bond: Met1640 (3.07), Carbon Hydrogen Bond: Thr1641 (2.39), Thr1641 (2.85)	Pi-Sigma: Ile1702 (2.57), Pi-Pi Stacked: Phe1706 (4.57), Phe1706 (4.18), Pi-Pi T-Shaped: Phe1674 (5.56)
Gallic acid	−3.163	−23.014	−16.94	−4.13	26.14	5.64	−0.53	−11.94	−1.35	−11.17	−23.73	Conventional Hydrogen Bond: Thr1641 (1.85)	Pi-Sulfur: Met1677 (5.56), Pi-Pi T-Shaped: Phe1706 (5.33), Pi-Alkyl: Met1640 (4.68)
**Potassium voltage-gated ion channel (KCNQ1; PDB: 5VMS)**													
Vitexin	−6.531	−47.301	−30.79	−10.14	−32.95	8.1	−4.04	−7.73	−1.83	37.71	−30.05	Conventional Hydrogen Bond: Arg507 (2.43), Gln42 (2.45), Gln42 (1.77), Glu48 (1.88), Lys383 (1.82), Ile384 (3.10), Carbon Hydrogen Bond: Pro44 (3.00), Glu48 (3.00), Gln510 (2.86), Glu48 (2.73), Glu115 (2.81)	Pi-Cation: Arg370 (4.57), Arg370 (3.81), Pi-Alkyl: Lys116 (4.93), Ile384 (3.91)
Orientin	−5.5	−49.421	−26.99	−8.49	−31.63	4.94	−4.45	−3.91	−0.13	37.36	−29.17	Conventional Hydrogen Bond: Lys517 (2.54), Lys116 (2.69), Glu115 (2.39), Glu115 (2.03), Ser363 (2.18), Asp51 (2.33), Gly114 (2.13), Gly114 (1.71), Carbon Hydrogen Bond: Glu115 (2.98)	Pi-Anion: Glu48 (4.78), Glu48 (4.61)
Gallic acid	−5.135	−26.826	−13.04	−2.43	−13.13	3.17	−2.45	−3.12	0	19.84	−17.34	Conventional Hydrogen Bond: Lys517 (2.35), Gln536 (2.77), Ser363 (1.77), Glu115 (2.57), Carbon Hydrogen Bond: Ala360 (2.68), Ala514 (2.83)	Attractive Charge: Lys517 (4.75), Pi-Cation: Lys517 (3.91), Pi-Alkyl: Lys517 (4.05)
Human β-1 adrenergic receptor (PDB: 7BTS)													
Orientin	−7.522	−52.002	−46.07	−16.78	−34.46	2.41	−3.87	−8.75	−2.54	42.51	−41.37	Conventional Hydrogen Bond: Asn1034 (2.29), Thr1058 (2.15), Val1120 (2.26), Asp1217 (2.08), Asp1217 (1.94), Carbon Hydrogen Bond: Arg1357 (3.02), Val1120 (2.77)	Pi-Pi Stacked: Trp1057 (5.62), Trp1057 (4.22), Pi-Alkyl: Arg1357 (4.94)
Vitexin	−6.611	−46.429	−44.36	−16.04	−23.38	1.27	−3.13	−8.78	−2.45	30.89	−38.79	Conventional Hydrogen Bond: Arg1038 (2.31), Arg1038 (2.79), Asn1034 (2.03), Ser1054 (1.82), Asp1217 (1.87), Carbon Hydrogen Bond: Arg1357 (3.07), Val1120 (3.09)	Pi-Pi Stacked: Trp1057 (5.69), Trp1057 (4.33), Pi-Alkyl: Arg1357 (4.73), Val1360 (5.45)
Gallic acid	−6.565	−27.204	−16.29	−3.85	−18.03	3.43	−1.85	−9.29	−0.36	34.7	−24.91	Conventional Hydrogen Bond: Phe1218 (2.03), Trp1364 (2.13), Asp1217 (2.56), Asn1363 (1.75), Carbon Hydrogen Bond: Gly1115 (2.74), Gly1115 (3.00)	Pi-Sigma: Val1360 (2.87)

Values are expressed as the mean ± standard deviation (SD), *n* = 3.

### 3.11 Computational cardiomyocyte models

We studied the effects of gallic acid at concentrations 10, 50, and 100 µM in different computational cardiomyocyte models to evaluate the membrane action potential, calcium transients during steady state, I_kr_ during steady-state transit, upstroke velocity (dV/dt_max_), and effective refractory period (ERP) at 1, 2, and 3 Hz, using the models given below:

#### 3.11.1 Human LV endocardium model

**FIGURE 10:**
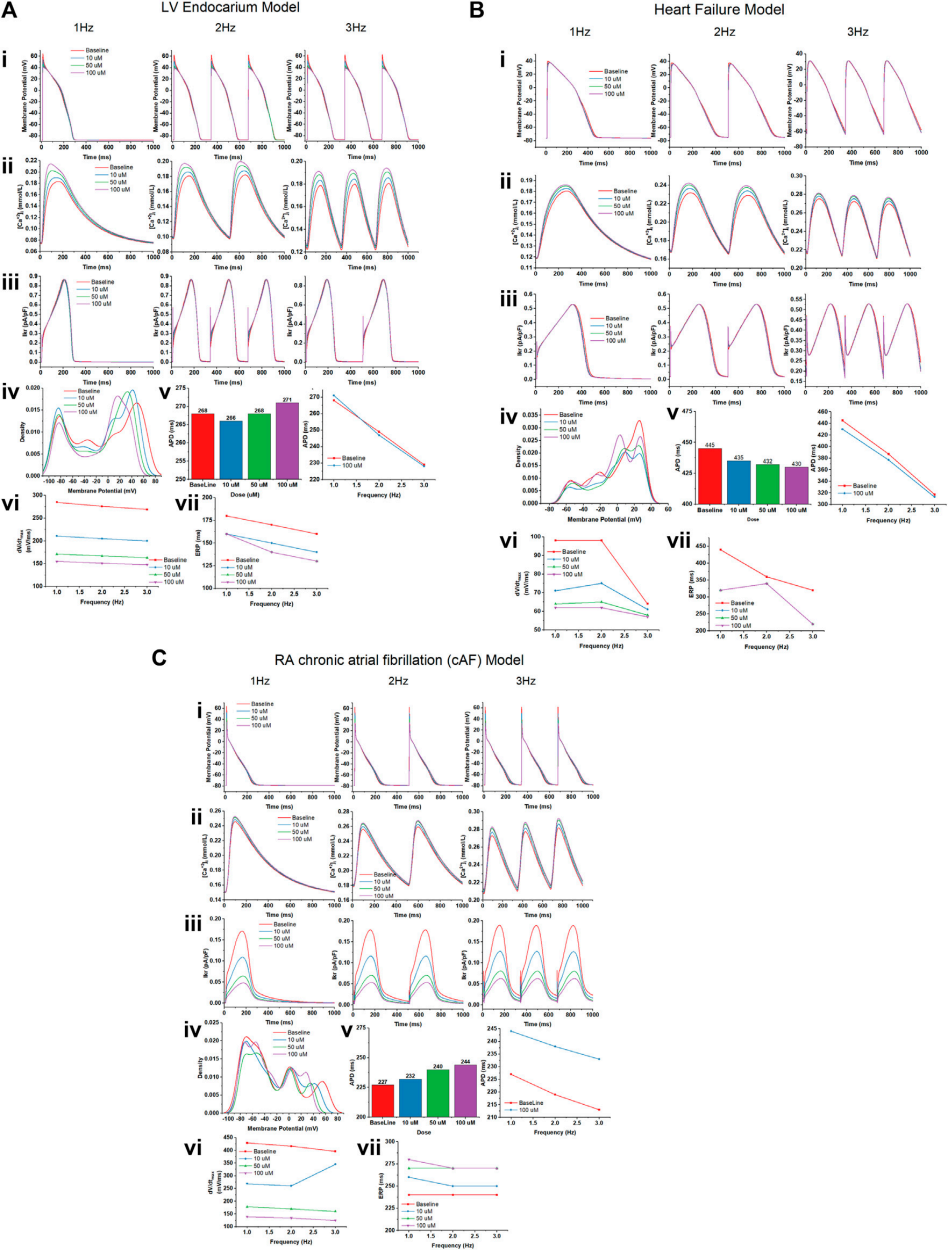
The effect of gallic acid on different computational cardiomyocyte models; **(A)** Human LV Endocardium model, **(B)** Human LV heart failure model, and **(C)** RA chronic atrial fibrillation model for electrophysiological studies. **(i)** Membrane action potential, **(ii)** calcium transients during steady state, and **(iii)** I_kr_ during steady-state pacing at pacing 1, 2, and 3 Hz in the absence (baseline) and presence of gallic acid (10, 50, and 100 µM). **(iv)** The density plot showed the distribution of the effect on action potential in the presence and absence of gallic acid. **(v)** The effect of gallic acid on action potential duration (APD) at 1, 2, and 3 Hz. **(vi)** The frequency dependence effect of gallic acid on **(vi)** upstroke velocity (dV/dt_max_) and **(vii)** effective refractory period (ERP) at pacing 1, 2, and 3 Hz.

Gallic acid reduces the membrane action potential amplitude dose-dependently in the human LV endocardium model (Figure 10Ai). The density plot showed that gallic acid reduces the action potential at 60 mV for a dose of 100 µM for pacing at 3 Hz (Figure 10Aii), indicating the blockade of I_Na_ current through the closing of sodium ion channels. Furthermore, we studied the effect of gallic acid on the calcium transit [Ca^+2^]_i_ and I_Kr_ transit, where a dose-dependent increase in amplitude was observed (Figures 10Aiii, Aiv). Gallic acid slightly prolonged the action potential duration (APD) by 2% (271 ms) at a dose of 100 µM compared to baseline. When cardiomyocytes were paced at different frequencies (1, 2, or 3 Hz), the APD-rate dependence was observed. Furthermore, findings from this study showed that gallic acid at a dose of 100 µM indicates the rate-dependent reduction of APD in cardiomyocytes (Figure 10Av). The rate-dependent reduction was observed for upstroke velocity (dV/dt_max)_ and ERP. Gallic acid slowed the upstroke velocity by reducing it up to 45% (155 ms) at 100 µM compared to baseline (285 ms), and the rate-dependent reduction of upstroke velocity was observed when cardiomyocytes were paced at different frequencies (Figure 10Avi). Similarly, gallic acid reduced ERP by 12.2% at 100 µM and showed a rate-dependent reduction at different frequencies. The rate-dependent reduction pattern of ERP was similar at 50 and 100 µM doses of gallic acid (Figure 10Avii).

#### 3.11.2 Human LV heart failure model

A similar response of gallic acid was observed for the human LV heart failure model. Gallic acid dose-dependently lowered the membrane action potential amplitude in the human LV heart failure model (Figure 10Bi). The density plot showed that gallic acid decreases the action potential at 50 mV for a dose of 100 µM for pacing at 3 Hz (Figure 10Bii). Furthermore, we studied the effect of gallic acid on the calcium transit and I_Kr_ transit, where a dose-dependent increase in amplitude was seen (Figures 10Biii, Biv). Gallic acid decreased the action potential duration by 4% (430 ms) at 1 Hz for a dose of 100 µM compared to baseline (445 ms). In contrast, gallic acid at a dose of 100 µM showed the rate-dependent reduction of APD in cardiomyocytes when paced at different frequencies (Figure 10Bv). The rate-dependence reduction was observed for upstroke velocity (dV/dtmax) and ERP. Gallic acid lowers the upstroke velocity by reducing it up to 38.3% (62 ms) at 100 µM compared to baseline (98 ms), and the rate-dependent reduction of upstroke velocity was observed when cardiomyocytes paced at different frequencies. Similarly, gallic acid reduced ERP by 27.2% at 100 µM and showed a rate-dependent reduction at different frequencies (Figure 10Bvi). Furthermore, there was a similar rate-dependent reduction pattern for ERP at doses of 10, 50, and 100 μM, and ERP increased at doses of 50 µM and decreased at doses of 100 µM (Figure 10Bvii).

#### 3.11.3 Human RA chronic atrial fibrillation (cAF) model

Our findings showed that gallic acid dose-dependently decreases the membrane action potential amplitude in the human RA cAF (Figure 10Ci). The density plot revealed that gallic acid lowers the action potential at 52 mV for a dose of 100 μM at 3 Hz (Figure 10Cii). Furthermore, we studied the effect of gallic acid on the calcium and I_Kr_ transit, where a dose-dependent increase in amplitude was observed (Figures 10Ciii, Civ). In this study, gallic acid prolonged the action potential duration by 7% (244 ms) at 1 Hz for a dose of 100 µM compared to the baseline (227 ms). Additionally, gallic acid at a dose of 100 µM exerted a rate-dependent reduction of APD in cardiomyocytes when paced at different frequencies (Figure 10Cv). Moreover, gallic acid slowed the upstroke velocity by reducing it up to 67.6% (139 ms) at 100 µM compared to baseline (429 ms), and the rate-dependent reduction of upstroke velocity was observed when cardiomyocytes paced at different frequencies (Figure 10Cvi). Similarly, gallic acid increased ERP by 15% at 100 µM and showed a rate-dependent steady state at 2 and 3 Hz. The rate-dependent increased pattern of ERP was similar at 50 and 100 µM doses of gallic acid (Figure 10Cvii).

## 4 Discussion

*Cucumis melo* L. seeds have been used to treat a wide range of illnesses by traditional healers (Mallik et al., 2013; Mukherjee et al., 2013; Rajasree et al., 2016; Soltani et al., 2017). In this investigation, Cm-EtOH was evaluated as a therapeutic agent for chronic cardiovascular disease (Yang et al., 2015). Our findings showed that Cm-EtOH controls calcium homeostasis during smooth muscle contractions, leading to vasorelaxant effects. In addition, this study investigated how Cm-EtOH contributes to muscle relaxation and how calcium ions contribute to muscle relaxation. Research findings showed that calcium ions are crucial for smooth muscle contraction and relaxation by ensuring cytosolic calcium homeostasis (Hall and Hall, 2020). In this respect, it is well known that the excitation-contraction pathway regulates cytosolic calcium homeostasis, which is essential for smooth muscle contraction and relaxation (Sharkey and MacNaughton, 2018). We tested the potential vasorelaxant effects of Cm-EtOH on endothelial preparations and denuded rat aortic preparations. Cm-EtOH and verapamil were found to exert similar vasorelaxant effects in both aortic preparations. Cm-EtOH was also comparable to verapamil in terms of relaxation when exposed to PE (1 µM), K^+^ (80 mM), and K^+^ (25 mM). Furthermore, Cm-EtOH inhibited calcium entry into cells, relieving smooth muscle spasms (Gilani et al., 2004). On the other hand, K^+^ (80 mM) depolarized the membrane action potential, thus increasing calcium inward current (Saqib and Janbaz, 2016), whereas Cm-EtOH repolarized membrane action potentials (Saqib and Janbaz, 2021) to relax smooth muscle and prevent current calcium entry (Wallace and Sharkey, 2018; Hall and Hall, 2020).

Sympathetic nerves regulate the tone of vascular smooth muscles. Stimulating of α1-adrenergic receptors converts the phosphatidylinositol into inositol-1,4,5-triphosphate (PIP_3_), which boosts cytosolic calcium through the endoplasmic reticulum and voltage-dependent calcium ion channels. Within this context, PE stimulates the sympathetic nervous system by activating the α1-adrenoceptors, which release calcium from the cytosol and calcium influx from the extracellular fluid, resulting in long-lasting depolarization action potentials in vascular smooth muscle. Our results showed that Cm-EtOH suppresses the spastic contractions caused by K^+^ (80 mM) and K^+^ (25 mM) at 5 mg/mL, indicating that blockade of calcium ion channels response, thus inhibiting calcium influx. Accordingly, angina and hypertension can be effectively treated with calcium channel blockers (Ghayur et al., 2007; Saqib and Janbaz, 2016).

**FIGURE 11:**
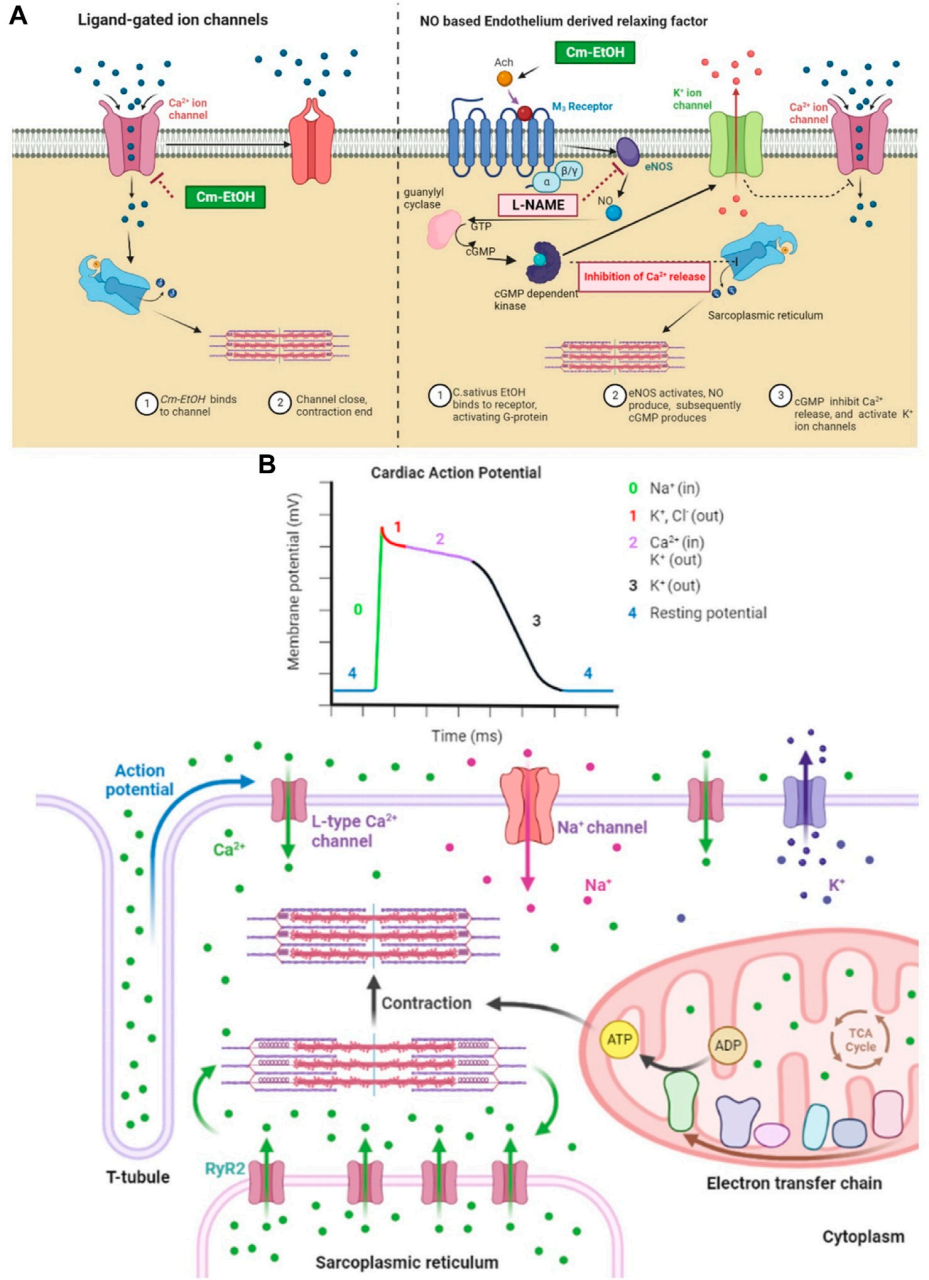
Mechanistic insight of Cm-EtOH on L type voltage-gated calcium ion channel, sodium ion channel, and NO-based endothelium-derived relaxing factor (EDRF). **(A)** Effect of Cm-EtOH on L type voltage-gated calcium ion channel and NO-based EDRF mechanism. **(B)** Role of sodium, calcium, and potassium ion channels in cardiac action potential with the increase in contraction of myocardial tissue.

Earlier, we showed that Cm-EtOH reduces intact and denuded aortic endothelium spastic contractions. However, PE-induced spasms in intact aortic endothelium could not relax in pretreated atropine preparations. The stimulation of muscarinic receptors causes nitric oxide (NO) release in vascular endothelium, which acts as a vasodilator. NO activates guanyl cyclase, which converts cGTP into cGMP. Thus, intracellular cGMP concentrations rise and trigger the cGMP-dependent protein kinase (PKG). The PKG intervenes with inositol 1,4,5-triphosphate receptor-associated cGMP kinase substrate and voltage-gated calcium ion channels, which lowers intracellular calcium levels (Figure 11) (Katzung, 2017).

The chronotropic and inotropic effects of Cm-EtOH on the paired atriums of rats were observed. Our findings also showed that Cm-EtOH reduces contraction force and causes a partial reduction in heart rate. Based on the results of Cm-EtOH on atrium preparation, it is assumed that the effects are attributed to cholinergic activity or to blocking calcium channels and adrenoceptors (Eschenhagen, 2018). In cardiac myocytes, atropine altered the inotropic and chronotropic actions of Cm-EtOH. These results suggest that Cm-EtOH works similarly to verapamil by activating cholinergic muscarinic receptors and blocking calcium channels (Saqib and Janbaz, 2021). Based on the results obtained on their aorta and atrium preparations, it is assumed that Cm-EtOH would have a hypotensive effect on anesthetized normotensive rats. It reduced the mean arterial, systolic, and diastolic blood pressure of rats under sedated conditions. The presence of non-inverted M_3_ receptors in rat aortic preparations and M_2_ receptors in cardiac tissues was associated with vasodilation after pretreatment with atropine (1 mg/kg). Moreover, Cm-EtOH may have antihypertensive effects via the inhibition of calcium channels.

Infarction is the primary cause of structural and functional changes in the myocardium. It is common in academia to inject isoproterenol into experimental animals to obtain a myocardial infarction (Shao et al., 2013; Meng et al., 2020; Zhou et al., 2020). The cardioprotective effects of Cm-EtOH were evaluated following chronic myocardial infarctions caused by ISO; results revealed that Cm-EtOH reduces ISO-induced MI and protects myocardial tissues. It has been demonstrated that ISO treatment significantly increases LHV weight, heart weight, and other MI measures. The serum biochemical parameters, including lipid profiles, cTnI, cTnT, LDH, CK-MB, CK, ALT, ACE AST, ANP, and BNP, showed a significant increase. Furthermore, the indices of ISO-induced cardiotoxicity were significantly reduced following pretreatment with Cm-EtOH like carvedilol and verapamil. Gene expression studies in cardiac tissues indicated that Cm-EtOH reduces the toxicity of isoprenaline.

**FIGURE 12:**
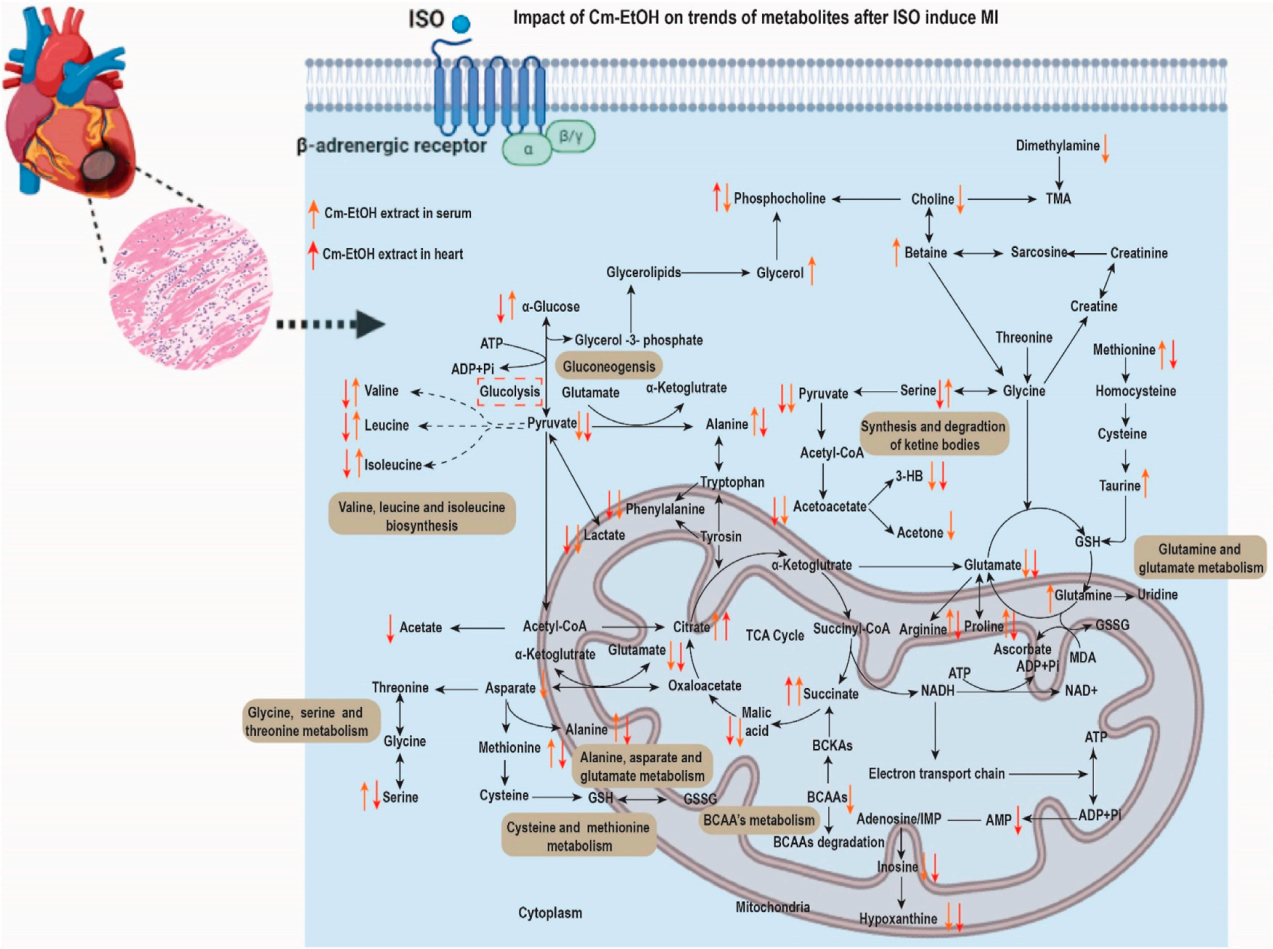
Cm.EtOH effects on serum and heart metabolites after ISO-induced MI.

In addition, Cm-EtOH increased eNOS3 mRNA expression and EDRF-mediated vasodilation. There was a significant reversal of the effects of Cm-EtOH, verapamil, and carvedilol on the inflammatory proteins MMP-9 and IL-6 and the cardiac proteins cTnT and ANP. Matrix metallopeptidase-9 (MMP-9) is believed to be the most critical factor in fibrosis and extracellular matrix degradation (Syed et al., 2016). The ISO group showed elevated expression of the MMP-9 gene due to autophagy and necrosis in cardiac tissues, but it was decreased by verapamil, carvedilol, and Cm-EtOH, suggesting a cardioprotective effect. The metabolomics analysis of serum and cardiac samples was performed to identify changes in endogenous metabolites between the groups. The alteration of putative metabolite biomarkers by Cm-EtOH resulted in hypotensive effects associated with ISO-induced anomalies in putative metabolite biomarkers. It has been proposed that several biochemical pathways, including nicotinamide, nicotinic acid, oxidative stress, pyruvate metabolism, and energy metabolism, are potential biomarkers (Figure 12). In this regard, numerous causes of hypertension are linked to the aforementioned biochemical pathways, including vascular endothelium dysfunction (Jiang et al., 2017).

The energy requirements of normal heart tissue are high; however, during myocardial infarction, these requirements become dramatically increased with oxygen and adenosine triphosphate (ATP) consumption. In this regard, ISO-induced MI requires inadequate energy for biosynthesis, resulting in excessive accumulation of free fatty acids and amino acids (Luan et al., 2015). It is commonly accepted that the production of mitochondrial ketone bodies increases following a decrease in myocardial and serum glucose levels during ischemia (Du et al., 2014). There is evidence that cardiac ischemia causes abnormalities in Kreb cycle intermediates. Furthermore, fumaric acid levels increased significantly in the ISO group, while citric acid and succinic acid levels decreased. The reduced blood glucose levels following MI indicate increased glycolysis in cardiac tissues (Hunter et al., 2016; Wende et al., 2017). The changes in glucose, lactate, pyruvate, and alanine concentrations indicate reduced glucose oxidation and increased glycolysis (Bin et al., 2017). These modified metabolites indicate that ISO-induced MI tissues exhibit altered energy metabolism. However, Cm-EtOH (150 mg/kg), carvedilol, and verapamil reverted this energy metabolism by improving the trends of glycolysis and Krebs cycle intermediate metabolites markers (Martínez et al., 2017), and also improved the ATP/ADP ratio, which enhances the availability of energy in myocardium tissues.

ISO administration altered serum and cardiac amino acid levels compared to the control group. This might be because cardiac tissues synthesize and degrade amino acids for energy (Martínez et al., 2017). MI and mitochondrial dysfunction are negatively correlated with glutamate. The rise in serum glutamate levels results in abnormalities in Krebs’ cycle, amino acid degradation, and ammonia detoxification (Nemutlu et al., 2015). Cm-EtOH regulates amino acids to promote cardio-protection. The alternation of serum branched-chain amino acids (BCAAs) indicates organ damage (Tso et al., 2014). In the event of an ISO-induced MI, an accumulation of BCAAs in injured organs could lead to a reduction in serum BCAA levels (Guo et al., 2016). When BCAAs accumulate in injured organs, they disrupt energy metabolism, mitochondrial function, and mTOR signaling, resulting in MI. Some amino acids (glutamine, methionine, and cysteine) are sensitive to ROS and reduce oxidative stress in injured organs (Bin et al., 2017). The amino acid methionine produces glutathione, which triggers antioxidant enzymes (Martínez et al., 2017). When ISO-induced hypoxanthine is produced in the endothelium, it causes oxidative stress and apoptosis, leading to endothelial dysfunction (Kim et al., 2017). ISO has also demonstrated myocardial remodeling by increasing proline levels (Zimmerli et al., 2008). Consequently, Cm-EtOH prevented myocardial ischemia by reducing oxidative stress and enhancing BCAAs, antioxidant amino acids, and nicotinamide levels. It can also detoxify the heart and improve amino acid metabolism.

Serotonin, a neuromodulator, and vasoconstrictor is obtained by the decarboxylation of 5-HTP. Several factors affect the production of epinephrine in the body, including serum 5-HTP levels. In this context, findings indicated that increased levels of nicotinamide, nicotinic acid, and 5-HTP are associated with hypertension and myocardial ischemia (Pörn-Ares et al., 2003). Increased levels of ceramide trigger protein kinases and apoptosis. When eNOS is inhibited, vascular endothelial cells experience oxidative stress, thus decreasing NO production and vascular dysfunction. Ceramide regulates the cyclooxygenase pathway by increasing arachidonic acid and thromboxane A2, which results in vasoconstriction and platelet aggregation (Liu et al., 2018). Several *in vitro* and gene expression experiments support the hypothesis that Cm-EtOH activates eNOS and enhances the relaxation of endothelium-dependent cells (Figure 12).

The heart function ensured with action potentials must propagate smoothly from the sinus node to the atria, then to the ventricles through the AV node. The cardiac conduction process refers to excitation propagation within the cell (intracellular conduction) and between cells (intercellular conduction). The cardiac conduction velocity is determined by the membrane depolarization rate responsible for excitation generation and the intercellular conductance for the propagation of excitations. In cardiac repolarization, the membrane potential returns to rest after depolarization (Yang et al., 2008; Zhang et al., 2019). In cardiac stimulation, the rate of membrane repolarization determines the length of action potential duration (APD) and effective refractory period (ERP), which represents the period during which a further action potential can occur (Sutanto et al., 2019). Moreover, in cardiac action potentials, ions flow across cell membranes to produce the currents that form the action potentials. Several factors influence the magnitude and modulation of individual currents, including transmembrane potential, depolarization time, and ligands (Zhang et al., 2019).

Moreover, because many channels are time- and voltage-dependent, drugs that target one channel may affect other channels by altering the trajectory of the action potential. The repolarization process is regulated by the delicate, yet dynamic, balance between inward and outward currents (Figure 11B). It has been observed that inward-flowing currents such as Na^+^ and L-type Ca^2+^ currents tend to depolarize the membrane and delay repolarization to prolong APD, whereas outward-flowing K^+^ currents such as *I*_*K1*_ *, I*_*Kur*_ *I*_*to*_ *, I*_*Kr*_ *,* and *I*_*Ks*_ tend to repolarize the membrane to shorten APD (Yang et al., 2008; Zhang et al., 2019). In this regard, I_Na_ plays an essential role in determining membrane depolarization velocity and intracellular conduction. The recovery from a blocked Na^+^ channel determines a steady-state Na^+^ channel block. Na^+^ channel blockers bind to (and block) open or inactivated Na^+^ channels, causing phasic changes in their extent during the action potential. Consequently, recovery from the block decreases, thus increasing the extent of the block (Eschenhagen, 2018).

## 5 Conclusion

In summary, findings from this investigation suggest that Cm-EtOH exerts vasorelaxant activity, negative chronotropic and ionotropic response, potent hypotensive through modulating NO-derived EDRF, and potent ability to prevent ISO-induced myocardial infarction in animals by reverting expressions of gene, biochemical markers, and serum and cardiac metabolites. In addition, our findings showed that profiling of metabolites in serum and cardiac tissue indicates that Cm-EtOH might exert cardioprotective effects in ISO-induced MI and reverse energy metabolism, amino acid metabolism, and oxidative stress. The effect of gallic acid in computational cardiomyocyte simulation reduced the action potential duration, upstroke velocity (dV/dt_max_), and effective refractory period (ERP) in different models, which could explain the cardioprotective effect of Cm-EtOH. These findings may explain the medicinal use of *C. melo* and provide mechanistic insight into its antihypertensive and cardioprotective activities. However, more detailed studies, that may involve human subjects, are required to establish the safety and efficacy of this plant.

## Data Availability

The original contributions presented in the study are included in the article/Supplementary Material, further inquiries can be directed to the corresponding authors.

## Abbreviations

ACN, Acetonitrile; ANP, Atrial natriuretic peptide; CVS, Cardiovascular system; CCB, Calcium channel blocker; Cm-EtOH, Hydroethanolic extract of *C. melo* seed; eNOS, Endothelial nitric oxide synthase; EDRF, endothelium-derived relaxing factor; FA, Formic acid; ISO, isoprenaline; L-NAME, Nω-nitro-l-arginine methyl ester; MM–GBSA, molecular mechanics energies combined generalized Born and surface area; MeOH, Methanol; MI, Myocardial infarction; MMP9, Matrix metallopeptidase 9; NO, nitric oxide; pKi, Predicted Log of Inhibition Constant (Ki); TFA, Trifluoroacetic acid.
