# Supplementary material for: Cardioprotective and hypotensive mechanistic insights of hydroethanolic extract of Cucumis melo L. kernels in isoprenaline-induced cardiotoxicity based on metabolomics and in silico electrophysiological models
Source: Front Pharmacol. 2024 Jan 29;14:1277594. doi: 10.3389/fphar.2023.1277594 (PMC10859416; doi:10.3389/fphar.2023.1277594)
Supplement: Supplementary file 1 [file DataSheet1.docx]

**Cardioprotective and hypotensive mechanistic insights of hydroethanolic extract of *Cucumis melo* L. kernels in isoprenaline-induce myocardial infraction based on metabolomics and in silico electrophysiological models.**

Muqeet Wahid^1^†, Fatima Saqib^1^†*, Ghulam Abbas^2^, Shahid Shah^3,^ Abdulrahman Alshammari^4^, Thamer H. Albekairi^4^, Anam Ali^1^, Muhammad Khurm^5^, Mohammad S. Mubarak^6^*

Table S1. Primers for qRT-PCR

| **Primer** | **Forward** | **Reverse** |
| --- | --- | --- |
| IL-6 | AGTTGCCTTCTTGGGACTGA | ACAGTGCATCATCGCTGTTC |
| ANP | ATACAGTGCGGTGTCCAACA | CGAGAGCACCTCCATCTCTC |
| cTnT | CAGAGAGGAGGAAGGTGCTG | TTCCCACGAGTTTTGGAGAC |
| eNOS3 | AACTAGACTGGGAGGGAGTCA | GGGCAGCAGGATGTCCTAATA |
| MMP-9 | CCCCCAACCTTTACCAGCTA | GGTCAGAACCGACCCTACAA |
| β- actin | AGCCATGTACGTAGCCATCC | CTCTCAGCTGTGGTGGTGAA |

**Table S2.** LC ESI-MS/MS identification of bioactive compounds in *C. melo* L hydroethanolic seed extract (Cm-EtOH).

| **Sr. No** | **Proposed compound** | **Empirical Formula** | **ESI-IT MS/MS (Ions)** | **Molecular Weight** | **Observed MS (m/z)** | **Calculated MS (m/z)** | **Error** | **Precursor type** | **Class** |
| --- | --- | --- | --- | --- | --- | --- | --- | --- | --- |
|  | Apigenin-7-*O*-Glucoside | C_21_H_20_O_10_ | 431, 270, 269, 268 | 432.40 | 431.0976 | 431.0988 | 2.8 | [M–H]^–^ | Flavonoid glycosides |
|  | Catechin | C_15_H_14_O_6_ | 291, 165, 139,123 | 290.27 | 291.0891 | 291.0889 | –0.7 | [M + H]^+^ | Flavonoid/Catechins |
|  | Chlorogenic acid | C_16_H_18_O_9_ | 353, 191, 173 | 354.31 | 353.0867 | 353.0861 | –1.7 | [M–H]^–^ | Alcohols and Polyols |
|  | Ellagic acid | C_14_H_6_O_8_ | 300.08, 283, 257.18,201, 207, 172 | 302.19 | 300.9963 | 300.9959 | –1.3 | [M–H]^–^ | Tannins |
|  | Epicatechin | C_15_H_14_O_6_ | 290, 289, 245, 179, 165, 151 | 290.27 | 289.0737 | 289.0726 | –3.8 | [M–H]^–^ | Catechin/ Flavonoids |
|  | Gallic acid | C_7_H_6_O_5_ | 169, 125, 85, 79 | 170.12 | 169.0152 | 169.0149 | –1.8 | [M–H]^–^ | Hydroxybenzoic Acid Derivatives |
|  | Naringenin | C_15_H_12_O_5_ | 271, 177, 151, 119, 107 | 272.25 | 271.0636 | 271.0645 | 3.3 |  | Flavanone |
|  | Naringenin-7-O-glucoside | C_21_H_22_O_10_ | 433, 272, 271, 177, 151, 119, 107 | 434.4 | 433.1163 | 433.1169 | 1.4 | [M–H]^–^ | Flavonoid glycosides |
|  | Orientin | C_21_H_20_O_11_ | 445, 417, 357, 327, 297, 269, 225, 171 | 448.38 | 447.0967 | 447.0963 | –0.9 | [M–H]^–^ | Flavone glucoside |
|  | Protocatechuic acid | C_7_H_6_O_4_ | 153, 109, 91, 80, 65 | 154.12 | 153.0173 | 153.0176 | 2.0 | [M–H]^–^ | Hydroxybenzoic acid derivatives |
|  | Quinic acid | C_7_H_12_O_6_ | 191,173, 171, 127, 93, 85 | 192.17 | 191.0596 | 191.0599 | 1.6 | [M–H]^–^ | carboxylic acid |
|  | Vitexin | C_21_H_20_O_10_ | 431, 341, 311, 283, 117 | 432.40 | 431.0985 | 431.0991 | 1.4 | [M–H]^–^ | flavone glucoside |
|  | β-sitosterol | C_29_H_50_O | 412.08, 391.33, 365.33, 352.33, 311.25, 297, 285.08, 255.08, 171 | 414.70 | 413.379 | 413.3793 | 0.7 | [M–H]^–^ | Phytosterols |

**Table S3. Precision validation of analytical method of the *C. melo* seed extracts**

| Analytes | Theoretical concentration  (µg mL^-1^) | Inter-day precision (*n* = 5) | | Intra-day precision (*n*=5) | |
| --- | --- | --- | --- | --- | --- |
|  |  | Measured concentration  (µg mL^-1^) | RSD % | Measured concentration  (µg mL^-1^) | RSD % |
| Stigmasterol | 100 | 98.88 ± 0.51 | 0.52 | 98.88 ± 0.93 | 0.94 |
| β-sitosterol | 100 | 98.69 ± 0.78 | 0.79 | 97.96 ± 1.79 | 1.82 |
| Umbelliferone | 100 | 98.72 ± 0.68 | 0.69 | 98.05 ± 1.59 | 1.62 |
| Caffeic acid | 100 | 99.88 ± 0.61 | 0.61 | 98.38 ± 1.32 | 1.34 |
| Rutin | 100 | 98.88 ± 1.54 | 1.56 | 98.59 ± 1.74 | 1.77 |
| Quercetin | 100 | 98.60 ± 0.83 | 0.84 | 98.46 ± 1.60 | 1.63 |

Measurements were conducted in triplicates, and results are expressed as the mean ± S.D. Percent coefficient of variation (% RSD); (SD/Mean) ×100.

**Table S4. Accuracy validation of analytical method of the *C. melo* seed extracts through % recovery method**

| **Analytes** | **Standard additions µg/mL** | **% Recovery** | | | **Mean± SD** | **RSD%** |
| --- | --- | --- | --- | --- | --- | --- |
|  |  | **Day 1** | **Day 2** | **Day 3** |  |  |
| β-sitosterol | 50 | 49.50 | 48.40 | 50.11 | 49.81 ± 0.94 | 1.90 |
|  | 100 | 97.90 | 98.97 | 99.12 | 99.01 ± 0.74 | 0.74 |
|  | 200 | 194.02 | 197.94 | 196.43 | 196.92 ± 2.12 | 1.08 |
| Chlorogenic acid | 50 | 48.25 | 47.85 | 49.51 | 49.36 ± 1.32 | 2.68 |
|  | 100 | 97.02 | 96.67 | 99.55 | 98.84 ± 1.89 | 1.91 |
|  | 200 | 196.32 | 196.28 | 199.44 | 198.04 ± 1.66 | 0.84 |
| Protocatechuic acid | 50 | 48.90 | 49.05 | 49.51 | 49.50 ± 0.84 | 1.69 |
|  | 100 | 98.33 | 97.98 | 99.55 | 98.80 ± 0.89 | 0.90 |
|  | 200 | 196.99 | 200.91 | 199.44 | 199.10 ± 1.48 | 0.74 |
| Orientin | 50 | 48.51 | 49.49 | 49.11 | 49.37 ± 0.60 | 1.21 |
|  | 100 | 99.66 | 97.98 | 97.87 | 99.32 ± 1.34 | 1.35 |
|  | 200 | 196.99 | 194.98 | 199.24 | 197.81 ± 1.85 | 0.93 |
| Gallic acid | 50 | 47.52 | 48.50 | 48.11 | 48.86 ± 1.36 | 2.79 |
|  | 100 | 97.51 | 97.98 | 98.72 | 98.50 ± 0.99 | 1.01 |
|  | 200 | 195.60 | 196.95 | 198.04 | 197.75 ± 1.56 | 0.79 |
| Vitexin | 50 | 48.25 | 47.85 | 49.11 | 48.92 ± 0.89 | 1.82 |
|  | 100 | 97.02 | 96.67 | 99.22 | 98.66 ± 1.78 | 1.80 |
|  | 200 | 196.32 | 196.28 | 198.10 | 198.19 ± 2.26 | 1.14 |

All values are the mean ± S.D.; measurements were conducted in triplicates. Percent relative of SD variation (% RSD); (SD/Mean) ×100.

**Table S5.** Heart and serum OPLS-DA models of metabolomes in between ISO and treated groups.

| **Model Name** | **Serum** | | **Heart** | |
| --- | --- | --- | --- | --- |
|  | **R^2^X** | **Q^2^** | **R^2^X** | **Q^2^** |
| Control | 0.986 | 0.999 | 0.972 | 0.979 |
| Verapamil | 0.981 | 0.998 | 0.975 | 0.986 |
| Carvedilol | 0.981 | 0.998 | 0.974 | 0.983 |
| Cm-EtOH | 0.972 | 0.997 | 0.803 | 0.988 |

**
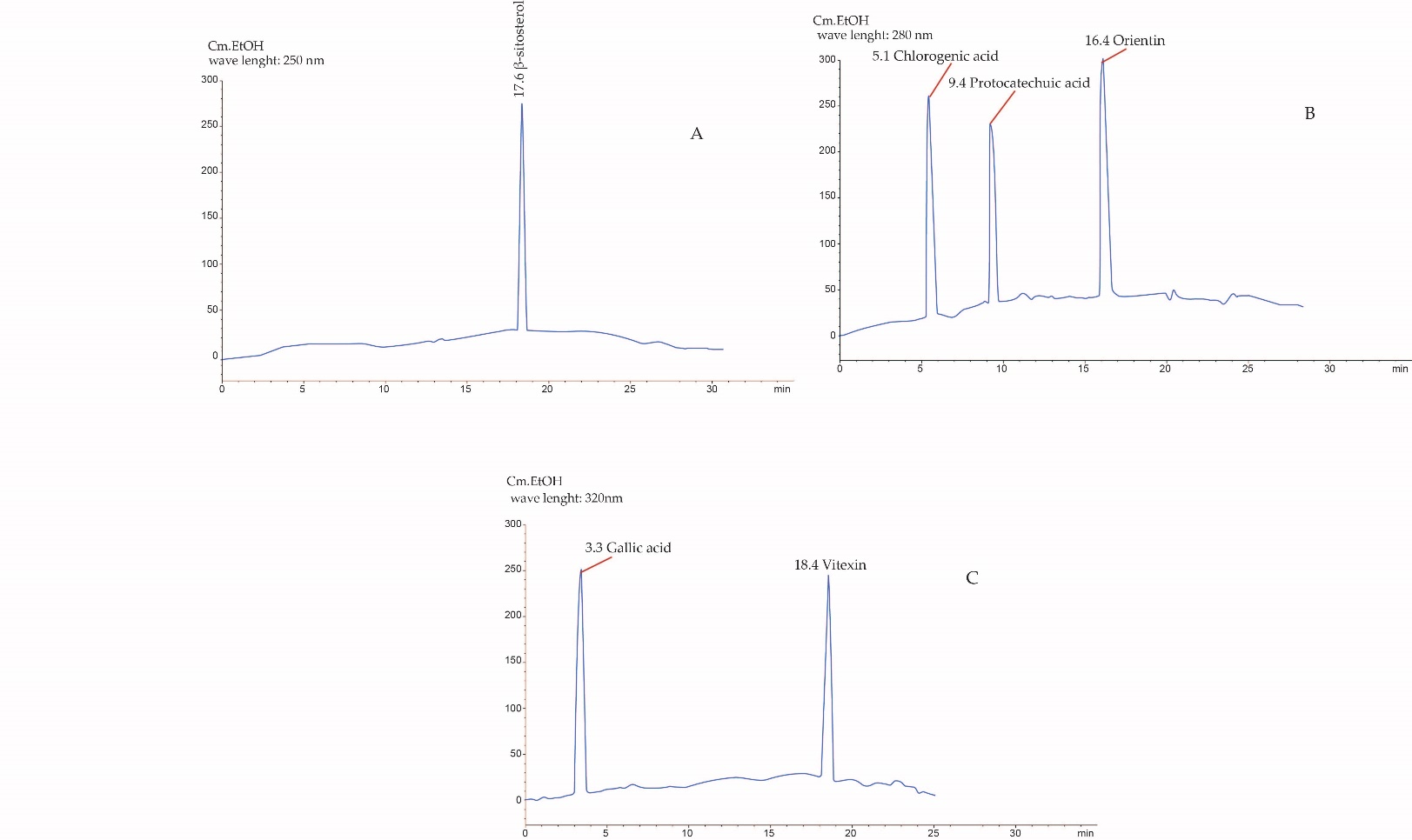
**

**Figure S1.** HPLC DAD-UV/Vis chromatograms of a *C. melo* seed extract at different wavelengths.


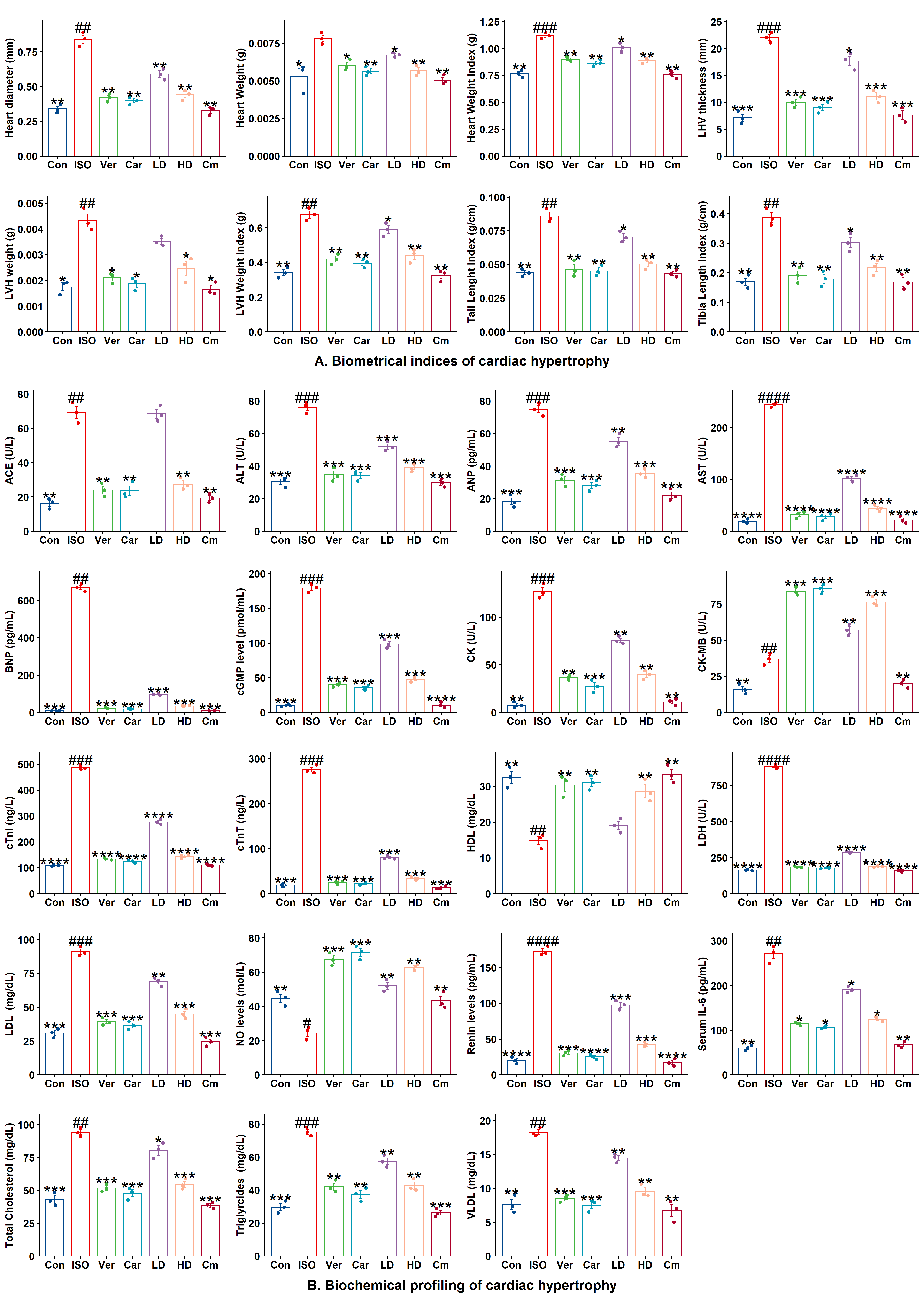


**Figure S2. S**erum biochemical changes in ISO-induced myocardial infarction tissues of ISO, carvedilol, verapamil, and Cm-EtOH.

Student t-tests were applied and compared to the ISO, whereas ISO is compared to the control; p < 0.05 was considered a significant value. Data are presented as the mean ± SD. n = 6, (****p < 0.0001, ***p < 0.001, **p < 0.01, and *p < 0.05, vs ISO; ####p < 0.0001, ###p < 0.001, ##p < 0.01, and #p < 0.05, versus the control). Con: Control, Car: Carvedilol (10 mg/kg); ISO: isoproterenol (5 mg/kg/day), Ver: Verapamil (10 mg/kg); LD: Low Dose (75 mg/kg); HD: High-Dose (150 mg/kg); Cm: Cm-EtOH (150 mg/kg).

**
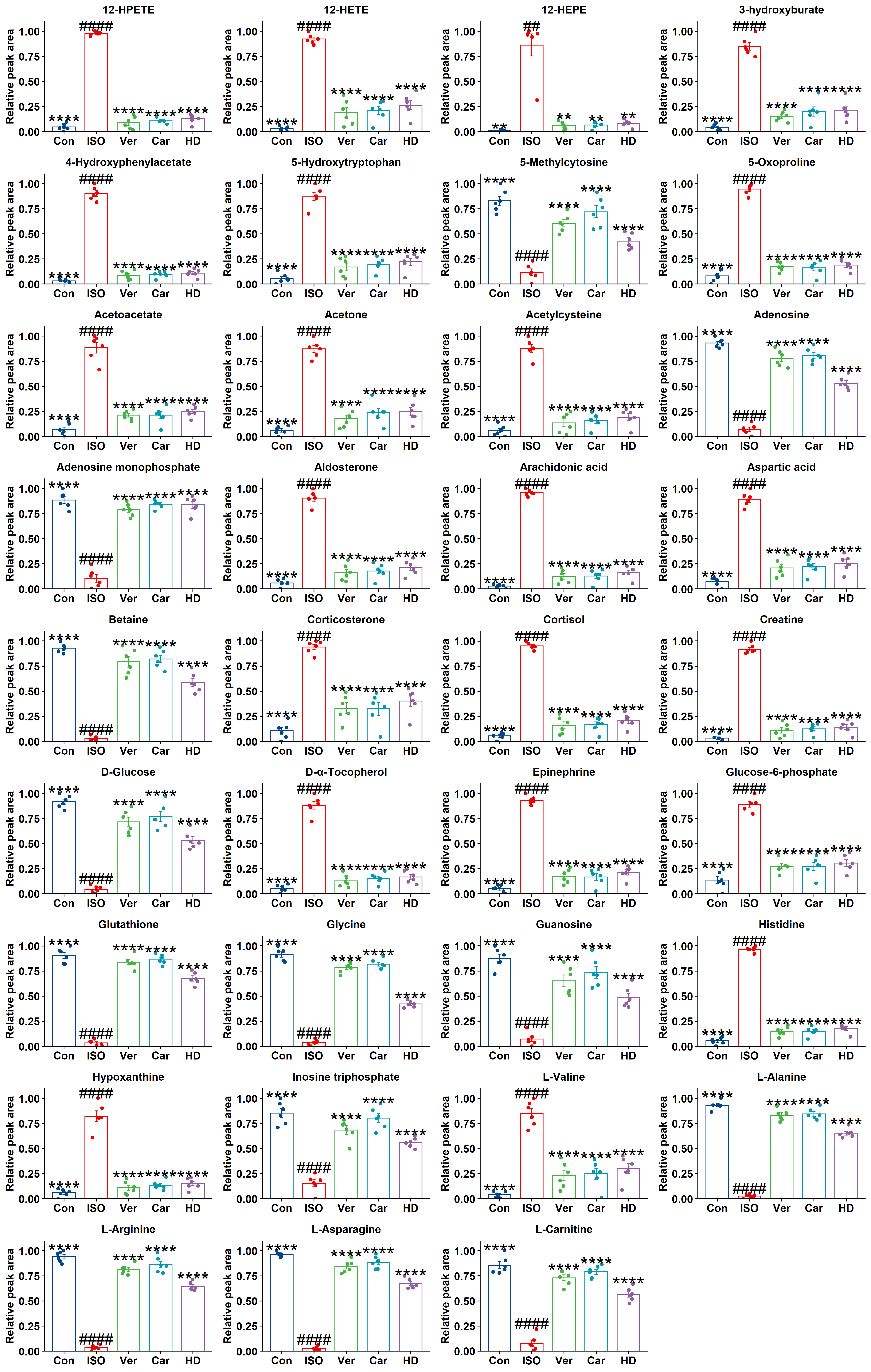
**

**Figure S3.** The metabolites levels of serum in ISO-induced myocardial infarction tissues of ISO, Carvedilol, Verapamil, Cm-EtOH.

Student t-tests were applied and compared to the ISO; whereas ISO compared to the control; p < 0.05 was considered a significant value. Data are presented as the mean ± SD. n = 6, (****p < 0.0001, ***p < 0.001, **p < 0.01, and *p < 0.05, vs ISO; ####p < 0.0001, ###p < 0.001, ##p < 0.01, and #p < 0.05, versus the control). Con: Control, Car: Carvedilol (10 mg/kg); ISO: isoproterenol (5 mg/kg/day), Ver: Verapamil (10 mg/kg); LD: Low Dose (75 mg/kg); HD: High-Dose (150 mg/kg); Cm: Cm-EtOH (150 mg/kg).

**
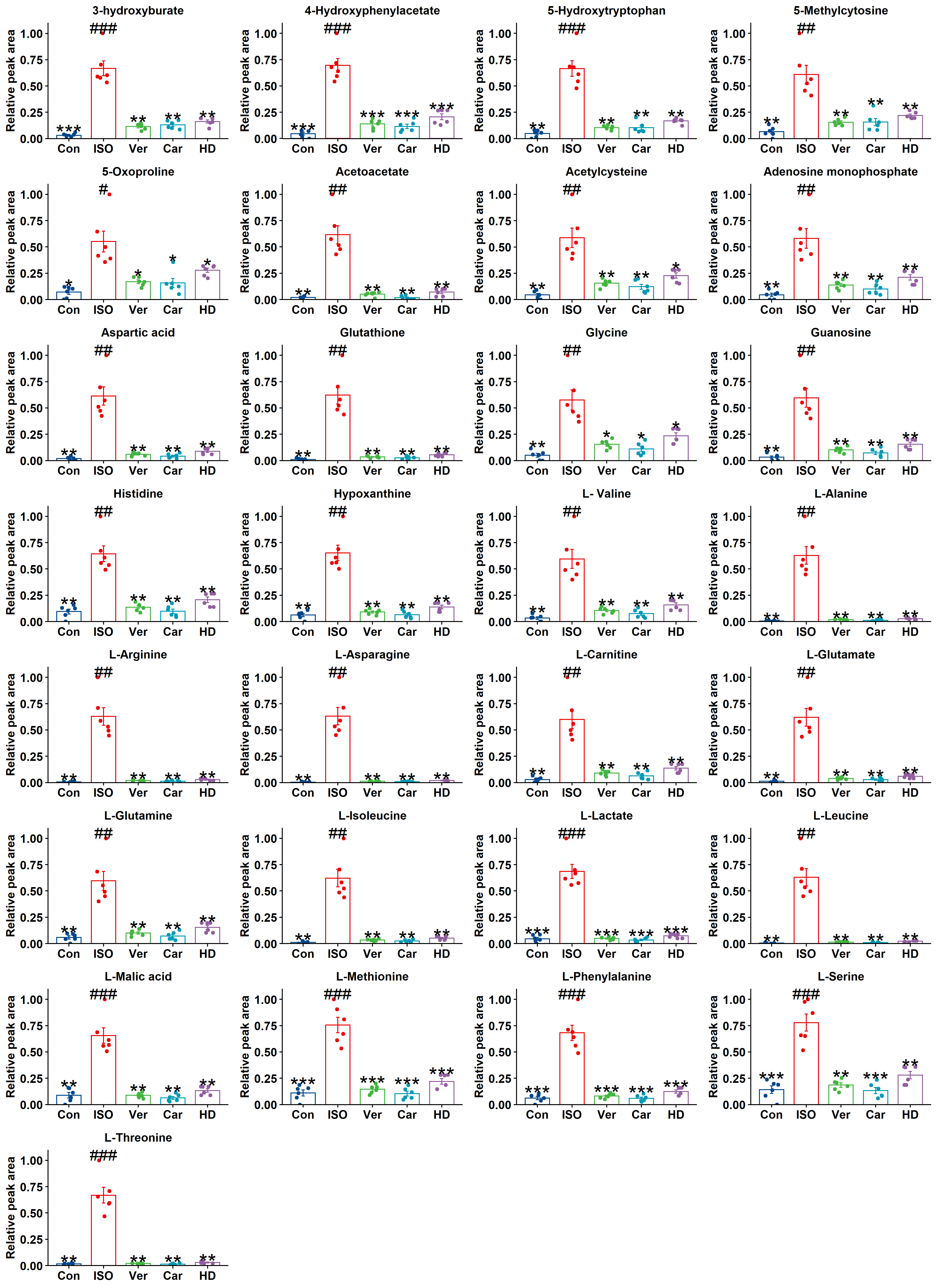
**

**Figure S4.** The metabolites levels of heart in ISO-induced myocardial infarction tissues of ISO, Carvedilol, Verapamil, Cm-EtOH.

Student t-tests were applied and compared to the ISO; whereas ISO compared to the control; p < 0.05 was considered a significant value. Data are presented as the mean ± SD. n = 6, (****p < 0.0001, ***p < 0.001, **p < 0.01, and *p < 0.05, vs ISO; ####p < 0.0001, ###p < 0.001, ##p < 0.01, and #p < 0.05, versus the control). Con: Control, Car: Carvedilol (10 mg/kg); ISO: isoproterenol (5 mg/kg/day), Ver: Verapamil (10 mg/kg); LD: Low Dose (75 mg/kg); HD: High-Dose (150 mg/kg); Cm: Cm-EtOH (150 mg/kg).


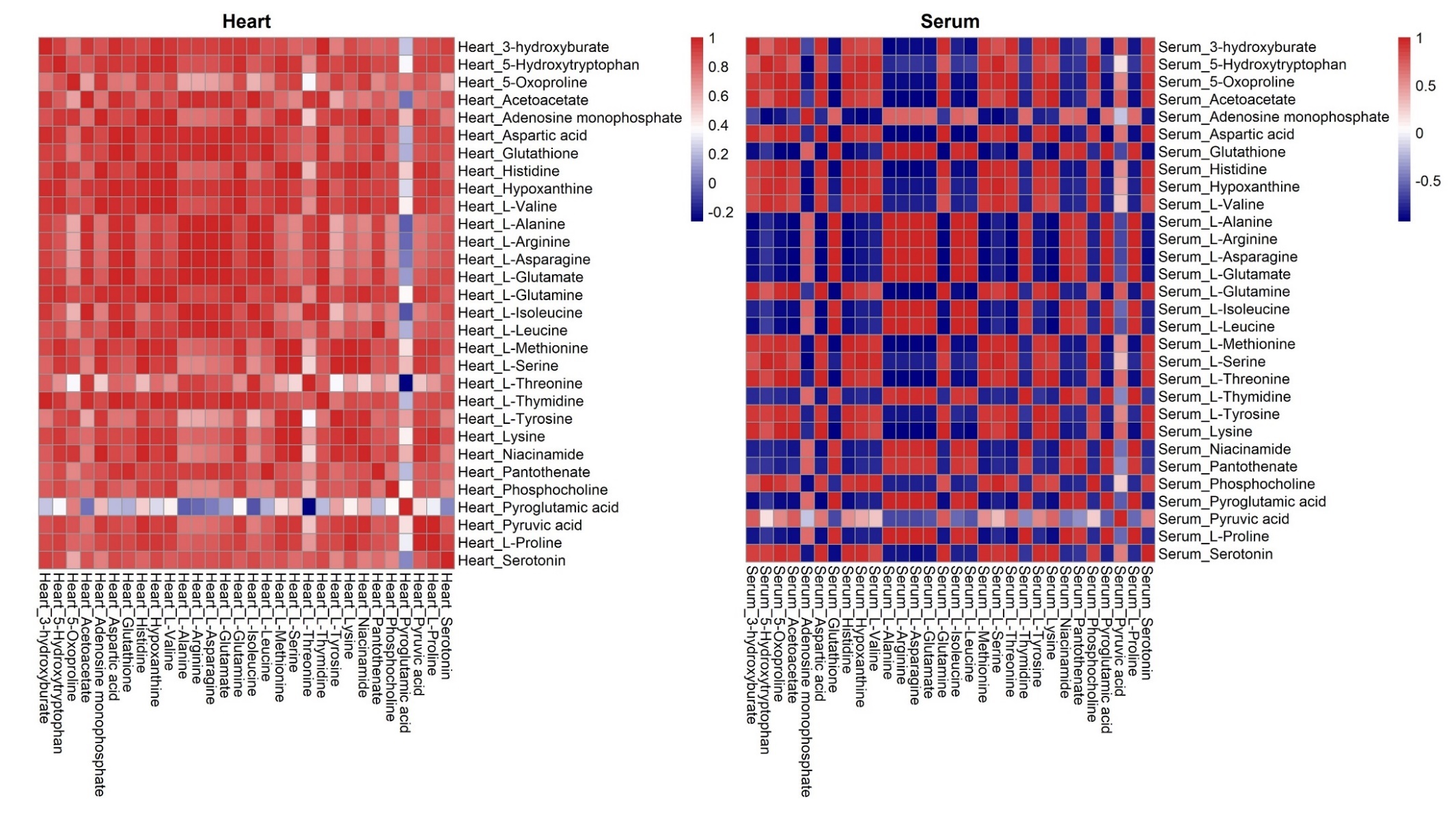


1. Correlation between the metabolites of serum and heart samples

**
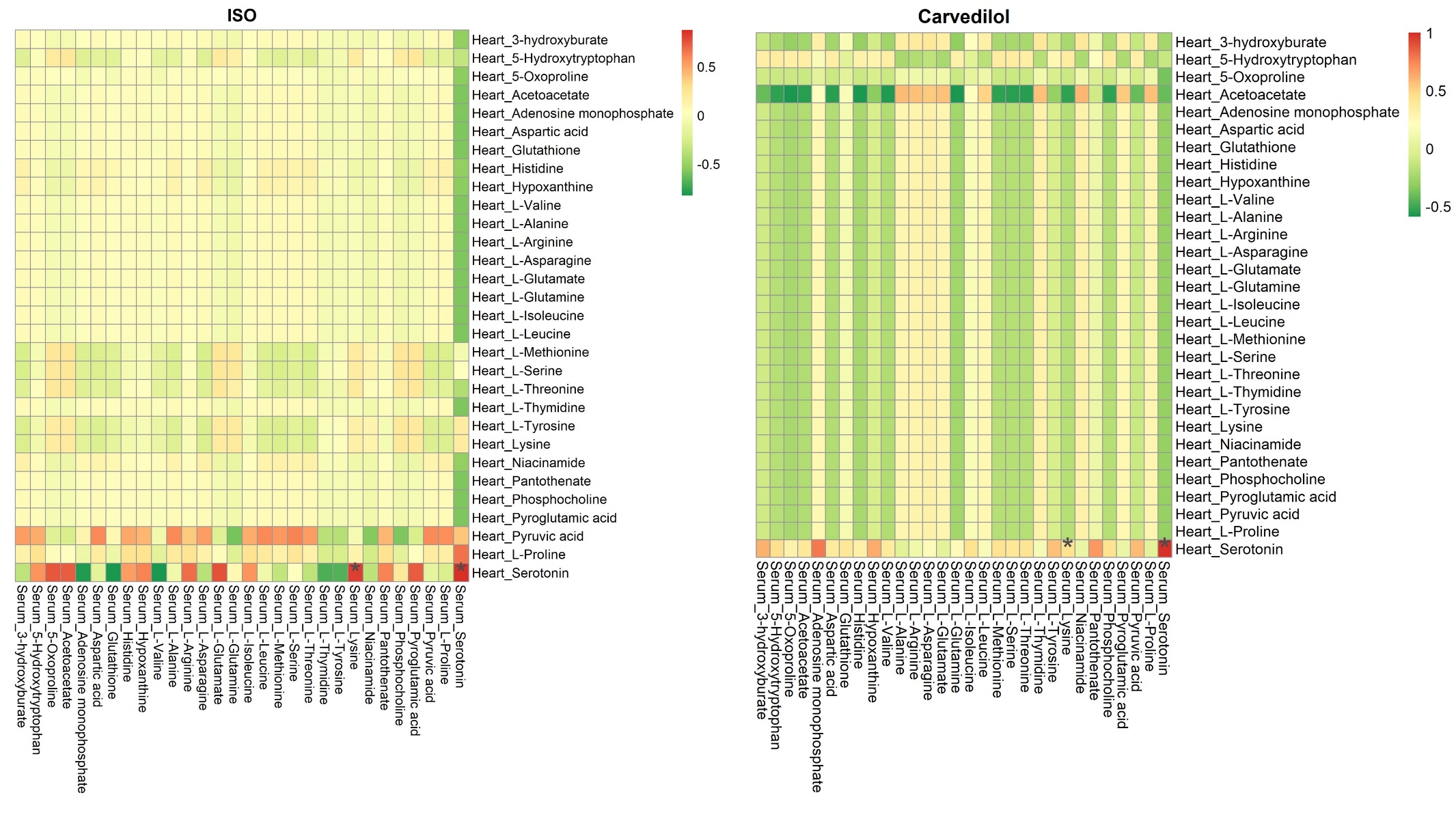
**

**
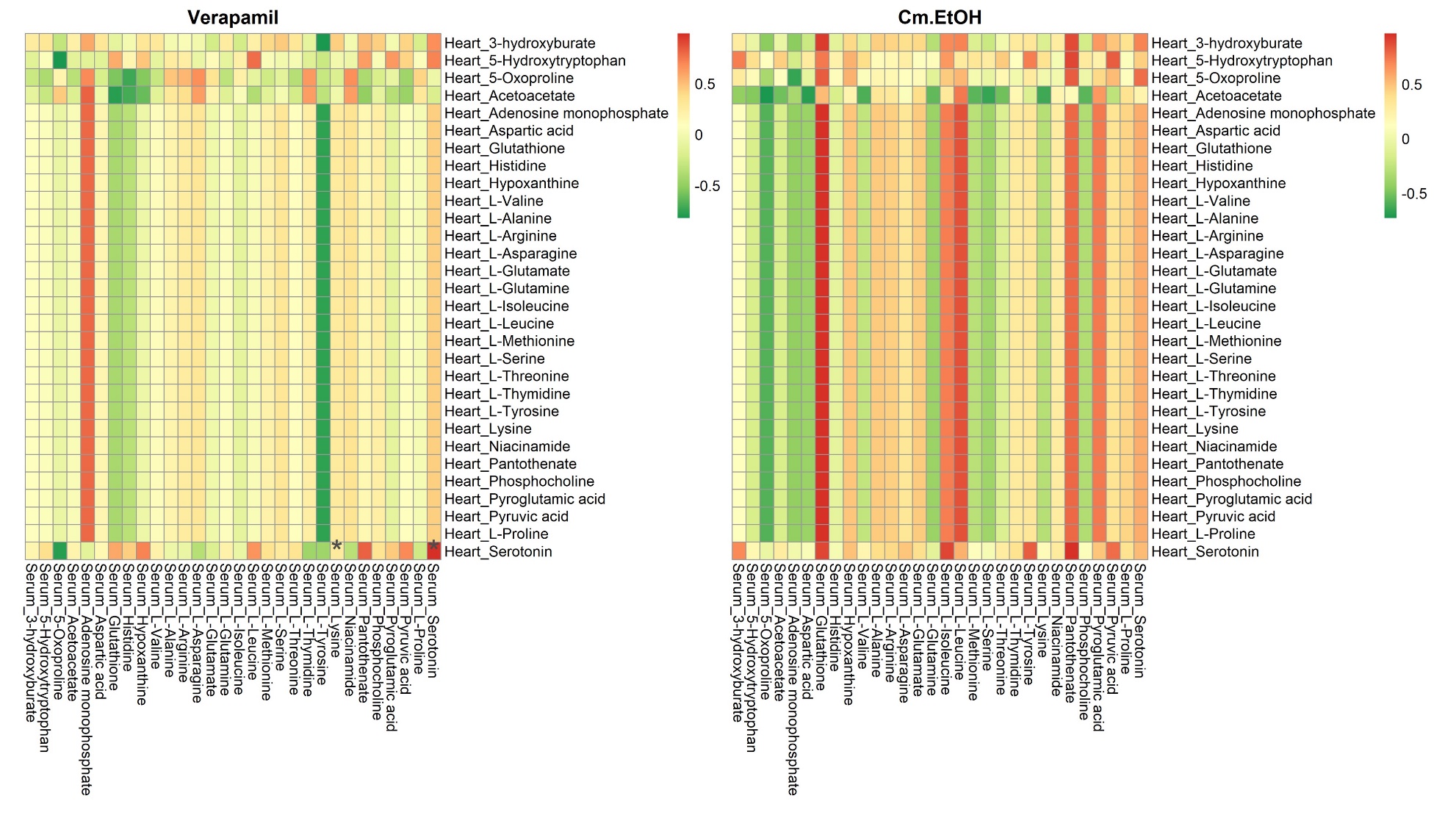
**

1. Correlation heatmap analysis of serum and heart metabolites in the control, ISO, verapamil, carvedilol group, and Cm-EtOH **(**150 mg/kg) groups.

**Figure S5.** The correlation heatmap analysis of serum and cardiac metabolites differed considerably after ISO-induced MI. **A.** Correlation between serum and cardiac metabolites and **B.** Analysis of the correlation between serum and cardiac metabolites for the control, ISO, verapamil, Carvedilol, and Cm-EtOH (150 mg/kg) groups.
